# Characterization of organic nitrogen in aerosols at a forest site in the southern Appalachian Mountains

**DOI:** 10.5194/acp-18-6829-2018

**Published:** 2018-05-16

**Authors:** Xi Chen, Mingjie Xie, Michael D. Hays, Eric Edgerton, Donna Schwede, John T. Walker

**Affiliations:** 1 National Risk Management Research Laboratory, Office of Research and Development, U.S. Environmental Protection Agency, Research Triangle Park, North Carolina 27711, USA; 2 Oak Ridge Institute for Science and Education (ORISE), National Risk Management Research Laboratory, Office of Research and Development, U.S. Environmental Protection Agency, Research Triangle Park, North Carolina 27711, USA; 3 Atmospheric Research and Analysis, Inc., Cary, NC 27513, USA; 4 National Exposure Research Laboratory, Office of Research and Development, U.S. Environmental Protection Agency, Research Triangle Park, North Carolina 27711, USA

## Abstract

This study investigates the composition of organic particulate matter in PM_2.5_ in a remote montane forest in the southeastern US, focusing on the role of organic nitrogen (N) in sulfur-containing secondary organic aerosol (nitrooxy-organosulfates) and aerosols associated with biomass burning (nitro-aromatics). Bulk water-soluble organic N (WSON) represented ~ 14% *w/w* of water-soluble total N (WSTN) in PM_2.5_ on average across seasonal measurement campaigns conducted in the spring, summer, and fall of 2015. The largest contributions of WSON to WSTN were observed in spring (~ 18% *w/w*) and the lowest in the fall (~ 10% *w/w*). On average, identified nitro-aromatic and nitrooxy-organosulfate compounds accounted for a small fraction of WSON, ranging from ~ 1% in spring to ~ 4% in fall, though were observed to contribute as much as 28% *w/w* of WSON in individual samples that were impacted by local biomass burning. The highest concentrations of oxidized organic N species occurred during summer (average of 0.65 ng N m^−3^) along with a greater relative abundance of higher-generation oxygenated terpenoic acids, indicating an association with more aged aerosol. The highest concentrations of nitro-aromatics (e.g., nitrocatechol and methyl-nitrocatechol), levoglucosan, and aged SOA tracers were observed during fall, associated with aged biomass burning plumes. Nighttime nitrate radical chemistry is the most likely formation pathway for nitrooxy-organosulfates observed at this low NO_*x*_ site (generally < 1 ppb). Isoprene-derived organosulfate (MW216, 2-methyltetrol derived), which is formed from isoprene epoxydiols (IEPOX) under low NO_*x*_ conditions, was the most abundant individual organosulfate. Concentration-weighted average WSON / WSOC ratios for nitro-aromatics + organosulfates + terpenoic acids were 1 order of magnitude lower than the overall aerosol WSON / WSOC ratio, indicating the presence of other uncharacterized higher-N-content species. Although nitrooxy-organosulfates and nitro-aromatics contributed a small fraction of WSON, our results provide new insight into the atmospheric formation processes and sources of these largely uncharacterized components of atmospheric organic N, which also helps to advance the atmospheric models to better understand the chemistry and deposition of reactive N.

## 1 Introduction

There is extensive evidence showing that boreal and temperate forests are affected by anthropogenic activities, both industrial and agricultural. Such activity results in unprecedented quantities of reactive nitrogen (N) being released into the atmosphere, subsequently altering global nitrogen and carbon (C) biogeochemical cycles (Bragazza et al., 2006; Doney et al., 2007; Ollinger et al., 2002; Magnani et al., 2007; Neff et al., 2002a, b; Pregitzer et al., 2008). Nitrogen enters natural ecosystems through atmospheric deposition and biological fixation and is mainly lost through leaching and gaseous fluxes back to the atmosphere (Hungate et al., 2003). The atmospheric deposition of N to terrestrial ecosystems may lead to soil and aquatic acidification, nutrient imbalance and enrichment, plant damage, and microbial community changes, as well as loss of biodiversity (Bobbink et al., 1998; Magnani et al., 2007; Lohse et al., 2008; Simkin et al., 2016).

In the United States, the deposition of atmospheric pollutants including N is monitored by the National Atmospheric Deposition Program (NADP) and the EPA’s Clean Air Status and Trends Network (CASNET). However, these networks focus only on inorganic N species (e.g., ${\text{NH}}_{3}\;∕\;{\text{NH}}_{4}^{+}$ and ${\text{HNO}}_{3}\;∕\;{\text{NO}}_{3}^{-}$). Recent studies shed light on the importance of organic N deposition, which is not routinely measured in national networks. On a global basis, organic N may contribute ~ 25% of the total N deposition (Gonzalez Benitez et al., 2009; Jickells et al., 2013; Kanakidou et al., 2012; Keene et al., 2002; Neff et al., 2002a; Zhang et al., 2012). Although ubiquitous, widespread routine monitoring of organic N in the atmosphere is inhibited due to difficulties in sampling (Walker et al., 2012) and inability to fully speciate the wide range of constituents that make up this large pool of atmospheric N (Altieri et al., 2009, 2012; Cape et al., 2011; Neff et al., 2002a; Samy et al., 2013). For these reasons, understanding of the sources, atmospheric chemistry, and deposition of organic nitrogen remains limited.

Atmospheric N from biogenic and anthropogenic emissions sources undergoes complex transformation processes and photochemical reactions. Consequently, the apportionment of atmospheric organic N to potential sources is challenging. However, such information is required to advance atmospheric N models applied to better understand the global N cycle. For example, Miyazaki et al. (2014) examined aerosols collected in a deciduous forest and found in the summer that water-soluble organic N (WSON) correlated positively with biogenic hydrocarbon oxidation, and during fall WSON in the coarse particle fraction was associated with primary biological emissions (e.g., emitted from soil, vegetation, pollen, and bacteria). Such patterns underscore the fact that atmospheric organic N measured in forested landscapes originates from a variety of sources that contribute differently across seasons.

Recent advancements have been made in the speciation of organic N in aerosol for some groups of compounds including amines, amino acids, and other nitrogenated functional groups such as organonitrates (Day et al., 2010; Place et al., 2017; Samy et al., 2013). Organic N in secondary aerosol and aerosols associated with biomass burning sources are areas of increasing interest, from both atmospheric chemistry and ecosystem exposure perspectives for which more information is needed. Studies of secondary organic aerosol (SOA) have identified a variety of nitrated organosulfate compounds (e.g., nitrooxy-organosulfates) in both chamber and ambient aerosol samples following isoprene and monoterpene oxidation. These compounds are either produced under high NO_*x*_ conditions or from nighttime NO_3_ radical chemistry (Surratt et al., 2006, 2007, 2008, 2010; Darer et al., 2011; Lin et al., 2013a; He et al., 2014; Worton et al., 2013). Potential SOA precursors such as unsaturated green leaf volatiles (GLVs) released by wounded plants (e.g., crop harvesting and insect attacks) may contribute substantially to the budget of biogenic SOA formation, especially in remote forests (Gomez-Gonzalez et al., 2008; Hamilton et al., 2013; Shalamzari et al., 2016). The detection of reaction products such as organosulfates and nitrooxy-organosulfates in ambient aerosols provides strong evidence of influence from anthropogenic sources (e.g., SO_2_ and NO_*x*_) interacting with biogenic precursors to form nitrogenated SOA (Chan et al., 2010; Lin et al., 2013a; Meade et al., 2016).

In addition to being present in sulfur-containing SOA, organic nitrogen, specifically nitro-aromatic compounds (e.g., nitrophenols and nitrocatechols), have been characterized as chemical tracers from biomass burning (e.g., wildland and prescribed smoke, bushfires, residential wood burning). This is in addition to levoglucosan, a widely used tracer of biomass burning (Iinuma et al., 2010, 2016; Kahnt et al., 2013; Kitanovski et al., 2012; Gaston et al., 2016). These nitrated compounds can form during the pyrolysis of plant biopolymers such as cellulose. Furthermore, as combustion by-products, these compounds are often defined as brown carbon (BrC) and are thus potentially light absorbing (Mohr et al., 2013; Liu et al., 2015). Presumably, nitro-aromatics could constitute a substantial portion of atmospheric organic N in aerosols collected in regions affected by biomass burning.

This study investigates the composition of organic particulate matter in a remote montane forest in the southeastern US, focusing on the role of organic N in sulfur-containing SOA and aerosols associated with biomass burning. The measurements target four groups of compounds: (1) nitro-aromatics associated with biomass burning; (2) organosulfates and nitrooxy-organosulfates produced from biogenic SOA precursors (i.e., isoprene, monoterpenes, and unsaturated aldehydes) interacting with anthropogenic pollutants; (3) terpenoic acids formed from monoterpene oxidation; and (4) organic molecular markers including methyltetrols, C-5 alkene triols, 2-methylglyceric acid, 3-hydroxyglutaric acid, and levoglucosan. Terpenoic acids and organic markers are included to assist in characterizing the extent of biogenic compound oxidation and atmospheric processing (i.e., aerosol aging) as well as contributions from biomass burning sources. Aerosol bulk chemical measurements are conducted to estimate total water-soluble organic N and C concentrations. The characterization of seasonal patterns in concentrations of organic N species and the assessment of potential sources and formation processes are emphasized.

## 2 Experimental methods and materials

### 2.1 Sampling site and atmospheric aerosol collection

The study was conducted at the US Forest Service Coweeta Hydrologic Laboratory, a 2185 ha experimental forest in southwestern North Carolina, USA (35°3′ N, 83°25′ W) near the southern end of the Appalachian Mountain chain. The climate is classified as maritime, humid temperate, with mean monthly temperatures ranging from 3.3 °C in January to 21.6 °C in July (Swift et al., 1988). Elevation ranges from 675 to 1592 m with a corresponding range in annual precipitation of 1800 to 2500 mm (Swank and Crossley, 1988). The vegetation is characterized as mixed coniferous–deciduous including oak, pines, and hardwoods (Bolstad et al., 1998). Atmospheric measurements were conducted in the lowest part of the basin (686 m), colocated with long-term measurements of air and precipitation chemistry conducted by the CASTNET and NADP networks, respectively.

The sampling site is 5 km west of Otto, NC (population 2500) and Highway 23 (Fig. S1 in the Supplement). Land to the north, west, and south of Coweeta is undeveloped forest. Typical rural development is present to the east of the site, consisting of houses and small-scale farming for hay and crop production including some scattered cow and horse pastures, which are small local ammonia (NH_3_) emission sources. The nearest metropolitan areas include Atlanta, Georgia (175 km southwest), Chattanooga, Tennessee (175 km west), Knoxville, Tennessee (110 km north–northwest), Asheville, North Carolina (100 km northeast), and Greenville, South Carolina (100 km southeast). The location of the sampling site within the context of NO_*x*_ and SO_2_ point sources in the eastern US is shown in the Supplement (Fig. S2). Only minor point sources are present within ~ 100 km of the site.

The study period summarized here comprises three seasonal intensives conducted during the spring, summer, and fall of 2015 as part of the Southern Appalachia Nitrogen Deposition Study (SANDS). Each campaign was conducted for approximately 3 weeks (21 May to 9 June, 6 to 25 August, 9 to 26 October). A high-volume Tisch TE-1000 (Tisch Environmental, Cleves, OH) dual-cyclone PM_2.5_ sampler operated at a flow rate of 230 L min^−1^ was set up on the ground to collect 24 h (started at 7:00 local time) integrated samples on pre-baked (550 °C for 12 h) quartz fiber (QF) filters (90mm; Pall Corporation, Port Washington, NY). Under some conditions, the 24 h integrated filter sampling technique may not fully retain all semi-volatile organic nitrogen compounds (Gonzalez Benitez et al., 2009). Field blanks were collected the same way except being loaded in the sampler without the pump switched on. A total of 58 ambient samples and 10 field blanks were obtained. Collected filter samples were transferred back to the laboratory in a cooler and stored in a freezer at −20 °C before chemical analysis.

### 2.2 Trace gas and meteorological measurements

During the spring 2015 campaign, NO_*x*_ concentrations were measured on a short tower (7 m above the ground) colocated with the CASTNET and high-volume PM samplers. NO_*x*_ concentrations were measured using a commercial NO-NO_2_-NO_*x*_ analyzer (model 42S, Thermo Environmental Instruments, Incorporated, Franklin, MA). Briefly, nitric oxide (NO) is measured directly on one channel by chemiluminescence. On a second channel, NO_2_ is converted to NO by a molybdenum catalyst heated to 325 °C, yielding the concentration of NO_*x*_ (NO + NO_2_). This approach may overestimate NO_*x*_ since other oxidized nitrogen gases such as HNO_3_, PAN, and HONO could also be reduced to NO on the heated molybdenum surface (Fehsenfeld et al., 1987; Williams et al., 1998; Zellweger et al., 2000). However, the use of an inlet filter and approximately 12 m of sample line between the atmospheric inlet and converter likely minimized the potential bias from HNO_3_. For subsequent campaigns, NO_*x*_ concentrations were estimated from a colocated NO_*y*_ analyzer. Similar to the NO_*x*_ instrument, NO_*y*_ and HNO_3_ were also measured using a modified model 42S NO-NO_2_-NO_*x*_ analyzer. The NO_*y*_ technique is described in detail by Williams et al. (1998). Briefly, total oxidized reactive nitrogen (NO_*y*_) is converted to NO using a molybdenum catalyst heated to 325 °C. On a second channel, a metal denuder coated with potassium chloride (KCl) is used to remove HNO_3_ before passing through a second molybdenum converter heated to 325 °C. The difference between the total NO_*y*_ measurement and the HNO_3_-scrubbed NO_*y*_ measurement is interpreted as HNO_3_. NO_*x*_ concentrations were estimated from the differences between measured NO_*y*_ and HNO_3_, which provided an upper-bound estimation as gaseous N-containing species were not excluded (e.g., PAN and organic nitrates). Hourly ozone concentrations were measured by CASTNET (US EPA, 2017) on a colocated 10 m tower. Hourly meteorological data were provided by CASTNET (US EPA, 2017) and the Forest Service (Miniat et al., 2015; Oishi et al., 2018), including temperature, relative humidity, solar radiation, and precipitation.

### 2.3 Chemical analysis

#### 2.3.1 Elemental and organic carbon analysis

A 1.5 cm^2^ QF punch was analyzed for elemental carbon (EC) and organic carbon (OC) using a thermo-optical transmittance (TOT) method (Sunset Laboratory Inc, Oregon, USA; Birch and Cary, 1996).

#### 2.3.2 Water-soluble species by ion chromatography (IC) and total organic carbon–total nitrogen (TOC-TN) analyzers

A second QF punch (1.5 cm^2^) from each sample was extracted with DI water (18.2 M Ω · cm; Milli-Q reference system, Millipore, Burlington, MA) in an ultrasonic bath for 45 min. The sample extract was filtered through a 0.2 μm pore size PTFE membrane syringe filter (Iso-disc, Sigma Aldrich, St. Louis, MO) before subsequent analyses.

Water-soluble organic carbon (WSOC) and total N (WSTN) concentrations were measured using a chemiluminescence method that included a total organic carbon analyzer (TOC-Vcsh) combined with a total nitrogen module (TNM-1; Shimadzu Scientific Instruments, Columbia, MD). For WSOC measurements, 25% phosphoric acid was mixed with sample extract (resulting in a 1.5% acid mixture) and sparged for 3 min to remove any existing carbonate and bicarbonate.

Inorganic species (${\text{NH}}_{4}^{+}$, ${\text{NO}}_{3}^{-}$, ${\text{NO}}_{2}^{-}$, and ${\text{SO}}_{4}^{2-}$) were analyzed using an ion chromatographer (IC; Dionex model ICS-2100, Thermo Scientific, Waltham, MA). The IC was equipped with guard (IonPac 2 mm AG23) and analytical columns (AS23) for anions. The samples were analyzed using an isocratic eluent mix of carbonate / bicarbonate (4.5 / 0.8 mM) at a flow rate of 0.25 mL min^−1^. Cations were analyzed by Dionex IonPac 2 mm CG12 guard and CS12 analytical columns; separations were conducted using 20 mM methanesulfonic acid (MSA) as eluent at a flow rate of 0.25 mL min^−1^. Multipoint (≥ 5) calibration was conducted using a mixture prepared from individual inorganic standards (Inorganic Ventures, Christiansburg, VA). A mid-level accuracy check standard was prepared from a certified standards mix (AccuStandard, New Haven, CT) for quality assurance and quality control purposes.

#### 2.3.3 UV–Vis light absorption analysis

Several studies have shown that methanol can extract aerosol OC at higher efficiencies than water and that a large fraction of light absorption in the near-UV and visible ranges is ascribed to water-insoluble OC (Chen and Bond, 2010; Liu et al., 2013; Cheng et al., 2016). In this study, a QF punch (1.5 cm^2^) was extracted with 5 mL methanol (HPLC grade; Thermo Fisher Scientific Inc.) in a tightly closed amber vial, sonicated for 15 min, and then filtered through a 0.2 μm pore size PTFE filter (Iso-disc; Sigma Aldrich, St. Louis, MO). The light absorption of filtered extracts was measured with a UV–Vis spectrometer over *λ* = 200–900 nm at 0.2 nm resolution (V660; Jasco Incorporated, Easton MD). The wavelength accuracy is better than ±0.3 nm; the wavelength repeatability is less than ±0.05 nm. A reference cuvette containing methanol was used to account for solvent absorption. The UV–Vis absorption of field blank samples was negligible compared to ambient samples, but used for correction nonetheless. For ease of analysis, the absorption at 365 nm referencing to absorption at 700 nm was used as a general measure of the absorption by all aerosol chromophore components (Hecobian et al., 2010).

#### 2.3.4 Analysis of isoprene and monoterpene SOA markers and anhydrosugars by GC-MS

Aliquots of each filter (roughly 1/4) were extracted by 10 mL of methanol and methylene chloride mixture (1 : 1, *v*/*v*) ultrasonically twice (15 min each). The total extract was filtered and concentrated to a final volume of ~ 0.5 mL. Next, extracts were transferred to a 2 mL glass vial and concentrated to dryness under a gentle stream of ultrapure N_2_ and reacted with 50 μL of N, O-bis(trimethylsilyl) trifluoroacetamide (BSTFA) containing 1% trimethylchlorosilane (TMCS), and 10 μL of pyridine for 3 h at 70 °C. After cooling down to room temperature, internal standards (mixture of 17.6 ng μL^−1^ acenaphthalene-d10 and 18.6 ng μL^−1^ pyrene-d4 mixed in hexane) and pure hexane were added. The resulting solution was analyzed by an Agilent 6890N gas chromatograph (GC) coupled with an Agilent 5975 mass spectrometer (MS) operated in the electron ionization mode (70 eV). An aliquot of 2 μL of each sample was injected in splitless mode. The GC separation was carried out on a DB-5 ms capillary column (30 m × 0.25 mm × 0.25 μm; Agilent Technologies, Santa Clara, CA). The GC oven temperature was programmed from 50 °C (hold for 2 min) to 120 °C at 30 °C min^−1^ then ramped at 6 °C min^−1^ to a final temperature of 300 °C (hold for 10 min). Linear calibration curves were derived from six dilutions of quantification standards. Anhydrosugars (levoglucosan) were quantified using authentic standard; 2-methyltetrols (2-methylthreitol and 2-methylerythritol) and C-5 alkene triols were quantified using meso-erythritol; other SOA tracers (e.g., hydroxyl dicarboxylic acid) were quantified using cis-ketopinic acid (KPA; refer to Table S1 in the Supplement). The species not quantified using authentic standards were identified by the comparison of mass spectra to previously reported data (Claeys, et al., 2004, 2007; Surratt et al., 2006; Kleindienst et al., 2007). Field blanks were collected and no contamination was observed for identified species.

#### 2.3.5 Analysis of organosulfates, terpenoic acids, and nitro-aromatics by high-performance liquid chromatography-electrospray ionization quadrupole time-of-flight mass spectrometry (HPLC-ESI-QTOF-MS)

Approximately 3–5 mL of methanol was used to ultrasonically extract (twice for 15 min) roughly half of each 90 mm QF sample. Internal standards (IS) were spiked onto each filter sample prior to extraction (refer to Tables S2, S3, and S4 for individual compounds and surrogate standards used for each group of compounds). Extracts were filtered into a pear-shaped glass flask (50 mL) and rotary evaporated to ~ 0.1 mL. The concentrated extracts were then transferred into a 2 mL amber vial that was rinsed with methanol two to three times. The final sample extract volume was ~ 500 μL prior to analysis. All the glassware used during the extraction procedure was pre-baked at 550 °C overnight. Extracted samples were stored at or below −20 °C prior to analysis and typically analyzed within 7 days.

An HPLC coupled with a quadrupole time-of-flight mass spectrometer (1200 series LC and QTOF-MS, model 6520; Agilent Technologies, Palo Alto, CA) was used for target compound identification and quantification. The QTOF-MS instrument was equipped with a multimode ion source operated in electrospray ionization (ESI) negative (−) mode. Optimal conditions were achieved under parameters of 2000 V capillary voltage, 140 V fragmentor voltage, 65 V skimmer voltage, 300 °C gas temperature, 5 L min^−1^ drying gas flow rate, and 40 psig nebulizer. The ESI-QTOF-MS was operated over the *m/z* range of 40 to 1000 at a 3 spectra s^−1^ acquisition rate. Target compound separation was achieved by a C18 column (2.1 × 100 mm, 1.8 μm particle size; Zorbax Eclipse Plus, Agilent Technologies) with an injection volume of 2 μL and flow rate of 0.2 mL min^−1^. The column temperature was kept at 40 °C, and gradient separation was conducted with 0.2% acetic acid (v : v) in water (eluent A) and methanol (eluent B). The eluent B was maintained at 25% for the first 3 min, increased to 100% in 10 min, held at 100% from 10 to 32 min, and then dropped back to 25% from 32 to 37 min, with a 3 min post-run time. During each sample run, reference ions were continuously monitored to provide accurate mass corrections (purine and HP-0921 acetate adduct; Agilent G1969-85001). Typically, the instrument exhibited 2 ppm mass accuracy. Tandem MS was conducted by targeting ions under collision-induced dissociation (CID) to determine parent ion structures. The Agilent software MassHunter was used for data acquisition (version B05) and for further data analysis (Qualitative and Quantitative Analysis, version B07). The mass accuracy for compound identification and quantification was set at ± 10 ppm. Calibration curves were generated from diluted standard compound mixtures. Recoveries of the extraction and quantification were performed by spiking known amounts of standards to blank QF filters. Then the spiked blank filters were extracted and analyzed the same way as ambient collected samples. The average recoveries of standard compounds are listed in the Supplement Table S5 and ranged from 75.2 ± 5.6 to 129.4 ± 4.2%. Isomers were identified for several compounds; no further separation was conducted, and combined total concentrations are reported in this study.

### 2.4 Source apportionment by positive matrix factorization

Positive matrix factorization (PMF) was used to identify potential sources of compounds measured at Coweeta. Here we use the PMF2 model (Paatero, 1998a, b) coupled with a bootstrap technique (Hemann et al., 2009), which has been applied in a number of previous studies (Xie et al., 2012, 2013, 2014). Briefly, PMF resolves factor profiles and contributions from a series of PM compositional data with an uncertainty-weighted least-squares fitting approach; the coupled stationary bootstrap technique generates 1000 replicated data sets from the original data set and each was analyzed with PMF. Normalized factor profiles were compared between the base case solution and bootstrapped solutions to generate a factor matching rate. The determination of the factor number was based on the interpretability of different PMF solutions (3–6 factors) and factor matching rate (> 50%). Detailed data selection criteria are presented in the Supplement.

## 3 Results and discussion

### 3.1 Meteorology, NO_*x*_, and O_3_

Statistics of atmospheric chemistry and meteorological measurements are summarized by season in Table 1. In general, the sampling site was humid and cool, even in the summer, with an average summer temperature of ~ 21 °C and RH of 82%. During the fall, much lower temperatures (~ 12 °C) and less humid conditions (RH = 78%) were observed. NO_*x*_ concentrations were generally less than 1ppb, which is considered typical for such a remote forest site removed from major emission sources.

[O_3_] (O_3_ concentration) was generally low (Table 1) with seasonal averages of 15 to 25 ppb. Historical seasonal [O_3_] levels over the past 5 years (2011 to 2015) are shown in the Supplement Figure S3. A spring maximum in [O_3_] is typically observed at this site, with lower concentrations during summer. Seasonal clustered back trajectories (Fig. S4 in the Supplement) suggest that during spring the Coweeta sampling site was under the influence of air masses transported from Atlanta urban areas. In addition, a spring maximum [O_3_] may be due to higher chemical consumption of O_3_ by reactive monoterpenes and sesquiterpenes emitted in the forest during summer. With observed relatively moderate summer temperatures and generally low [NO_*x*_], the site also experiences frequent cloud cover in summer, lowering the intensity of solar radiation, which may suppress ozone production relative to spring conditions. Additionally, deposition of O_3_ to the forest would be expected to peak during the summer when leaf area is greatest. O_3_ correlated positively with NO_*x*_ in summer and fall but not spring, indicating that O_3_ production might be relatively more VOC limited in spring than the other seasons in this region.

### 3.2 Bulk water-soluble organic nitrogen and carbon

**Figure 1.**
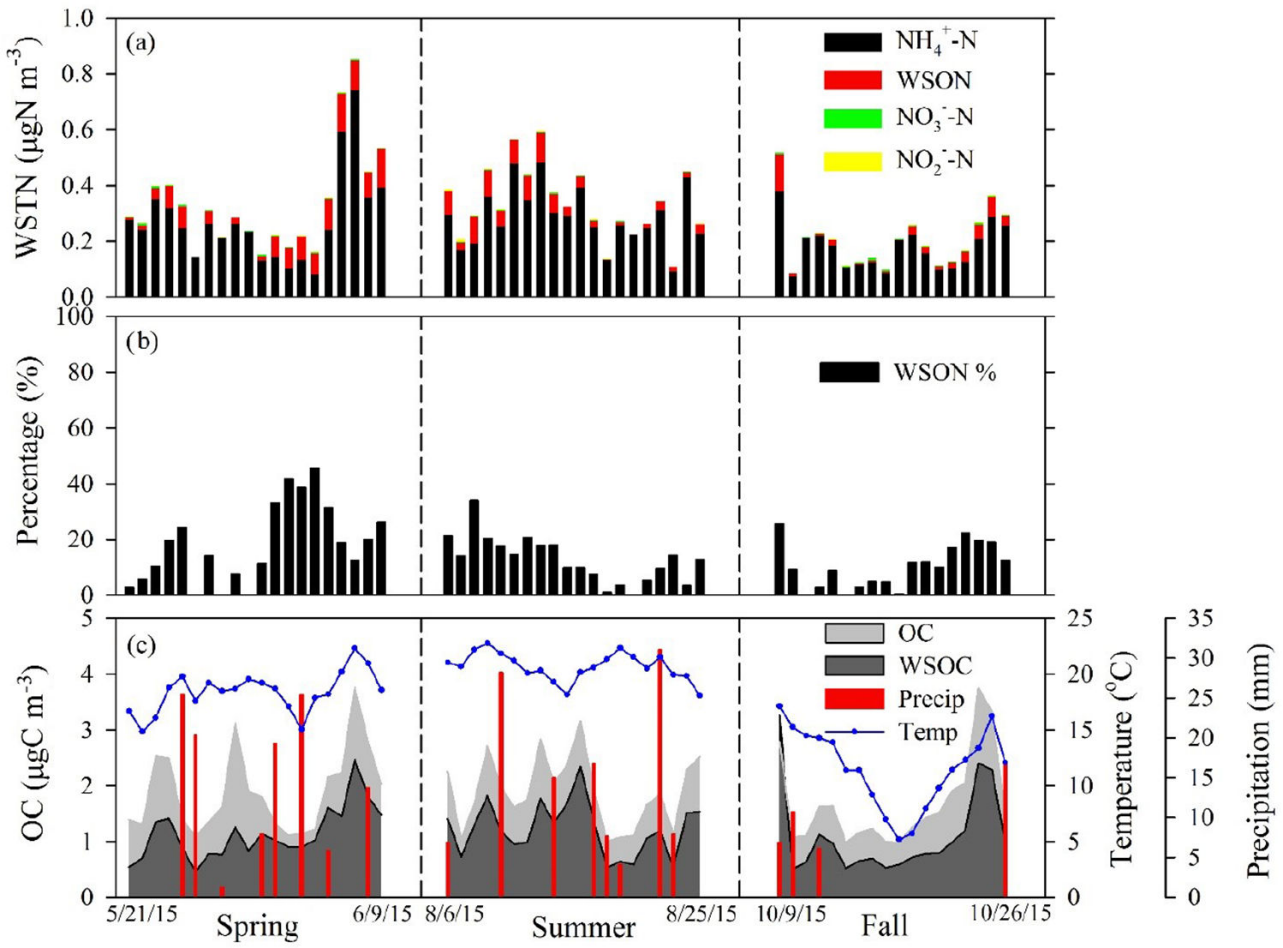
**(a)** Individual concentrations of nitrogen components to WSTN (${\text{NH}}_{4}^{+}$, ${\text{NO}}_{3}^{-}$, ${\text{NO}}_{2}^{-}$, and WSON). **(b)** Percent contribution of WSON to WSTN. **(c)** Time series of OC, WSOC, temperature, and precipitation. The start and end dates of each intensive sampling period are shown.

Water-soluble bulk organic N (WSON) was estimated as the difference between WSTN and the sum of the inorganic N species (${\text{NH}}_{4}^{+}$, ${\text{NO}}_{3}^{-}$, and ${\text{NO}}_{2}^{-}$). The measurement uncertainty of WSON was estimated to be ~ 30% from the error propagation of WSTN (2%), ${\text{NH}}_{4}^{+}$ (1%), ${\text{NO}}_{3}^{-}$ (1%), and ${\text{NO}}_{2}^{-}$ (1%). Nitrogen component contributions to WSTN are presented in Fig. 1a, which shows ${\text{NH}}_{4}^{+}$ as the most abundant component, contributing 85 ± 11% *w/w* to total WSTN mass. Typical ${\text{NH}}_{4}^{+}$ concentrations at the site were below 1.0 μg m^−3^ (with an average of 0.32 μg m^−3^), which is expected for such a remote site with no major local or regional NH_3_ sources. The oxidized inorganic N components (${\text{NO}}_{3}^{-}$ and ${\text{NO}}_{2}^{-}$) accounted for less than 2% *w/w* of WSTN measured. Such a small contribution of ${\text{NO}}_{3}^{-}$ to inorganic N (typically < 10% of inorganic N (${\text{NO}}_{3}^{-}$ + ${\text{NH}}_{4}^{+}$)) in PM_2.5_ is consistent with long-term CASTNET measurements at Coweeta. The average contribution of WSON to WSTN over the entire study period was 14 ± 11% *w/w*. This fraction reached a maximum of ~ 18% *w/w* in the spring (average) and a minimum of ~ 10% in the fall (average), exhibiting pronounced seasonal variability. Within individual samples (Fig. 1b), values ranged from near zero to 45%. Our study-wide average of 14% falls within the range of measurements at North American forest sites, including Duke Forest, North Carolina (~ 33%; Lin et al., 2010) and Rocky Mountain National Park (14–21%; Benedict et al., 2012). Moreover, the observed WSON contribution to WSTN in particles at Coweeta is consistent with a global estimated range of 10–39% (Cape et al., 2011).

WSOC accounted for roughly 62 ± 13% of OC throughout the entire study period with no significant seasonal variability. A time series of OC and WSOC along with temperature and precipitation is presented in Fig. 1c. On average, OC concentrations increased during warmer spring and summer seasons and decreased when the temperature decreased in fall. Concentrations of OC were positively correlated with temperature (*r* = 0.30, *p* < 0.05), presumably in response to emissions of biogenic precursors and the formation of secondary organic aerosols by photooxidation. Spring and summer were generally moist and warm with frequent precipitation (relative humidity presented in Table 1). Precipitation events corresponded to decreasing OC and WSOC concentrations, demonstrating the scavenging effect due to wet deposition.

Spearman rank correlation coefficients among measured species and meteorological variables as well as other gas-phase measurements are presented in Table 2 for each season (*p* < 0.01 for values in bold). As expected, ${\text{NH}}_{4}^{+}$ and ${\text{SO}}_{4}^{2-}$ tracked well over each season (*r* > 0.9, *p* < 0.01). ${\text{NH}}_{4}^{+}$ was mainly associated with ${\text{SO}}_{4}^{2-}$ given the fact that ${\text{NO}}_{3}^{-}$ and ${\text{NO}}_{2}^{-}$ were generally negligible compared to ${\text{SO}}_{4}^{2-}$. WSOC is often used as an SOA surrogate and accounts for a significant portion (62% *w/w*) of OC during all sampling periods. WSOC correlated strongly with OC over both summer and fall (*r* > 0.95, *p* < 0.01), but less so during spring (*r* = 0.74, *p* < 0.01). WSOC also positively correlated with WSON over spring and fall (*r* > 0.75, *p* < 0.01) but less so during summer (*r* = 0.5, *p* > 0.01). Note that both [WSOC] and [OC] were highest in the summer, which likely indicates higher biogenic emissions and SOA formation. However, the weak WSON–WSOC correlation suggests a variety of source contributions to WSON and WSOC over the different seasons. [EC] was negligible over the entire study except a modest spike at the end of October when wood burning was most likely the source. Details on this event are discussed in the subsequent sections. It is also noted that a stronger correlation of WSON with ${\text{NH}}_{4}^{+}$ than with ${\text{NO}}_{3}^{-}$ was observed, which might suggest a key role of reduced nitrogen in WSON formation (Cape et al., 2011; Jickells et al., 2013).

### 3.3 Nitro-aromatics

**Figure 2.**
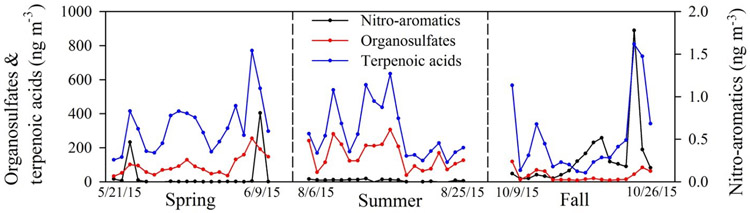
Time series of summed compound group concentrations of nitro-aromatics, organosulfates, and terpenoic acids.

**Figure 3.**
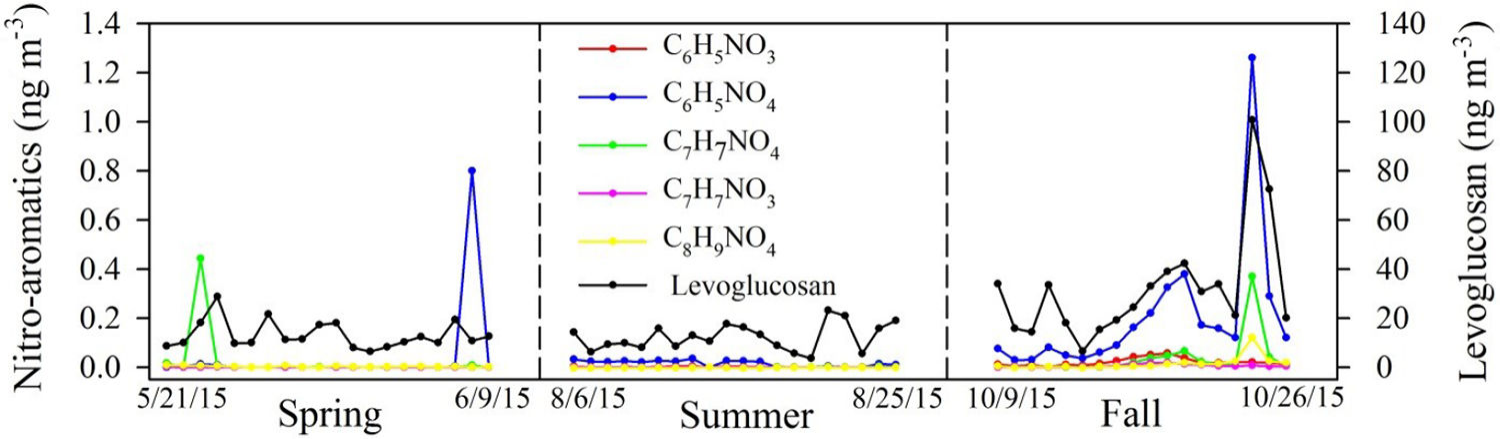
Time series of individual nitro-aromatic compounds and levoglucosan.

**Figure 4.**
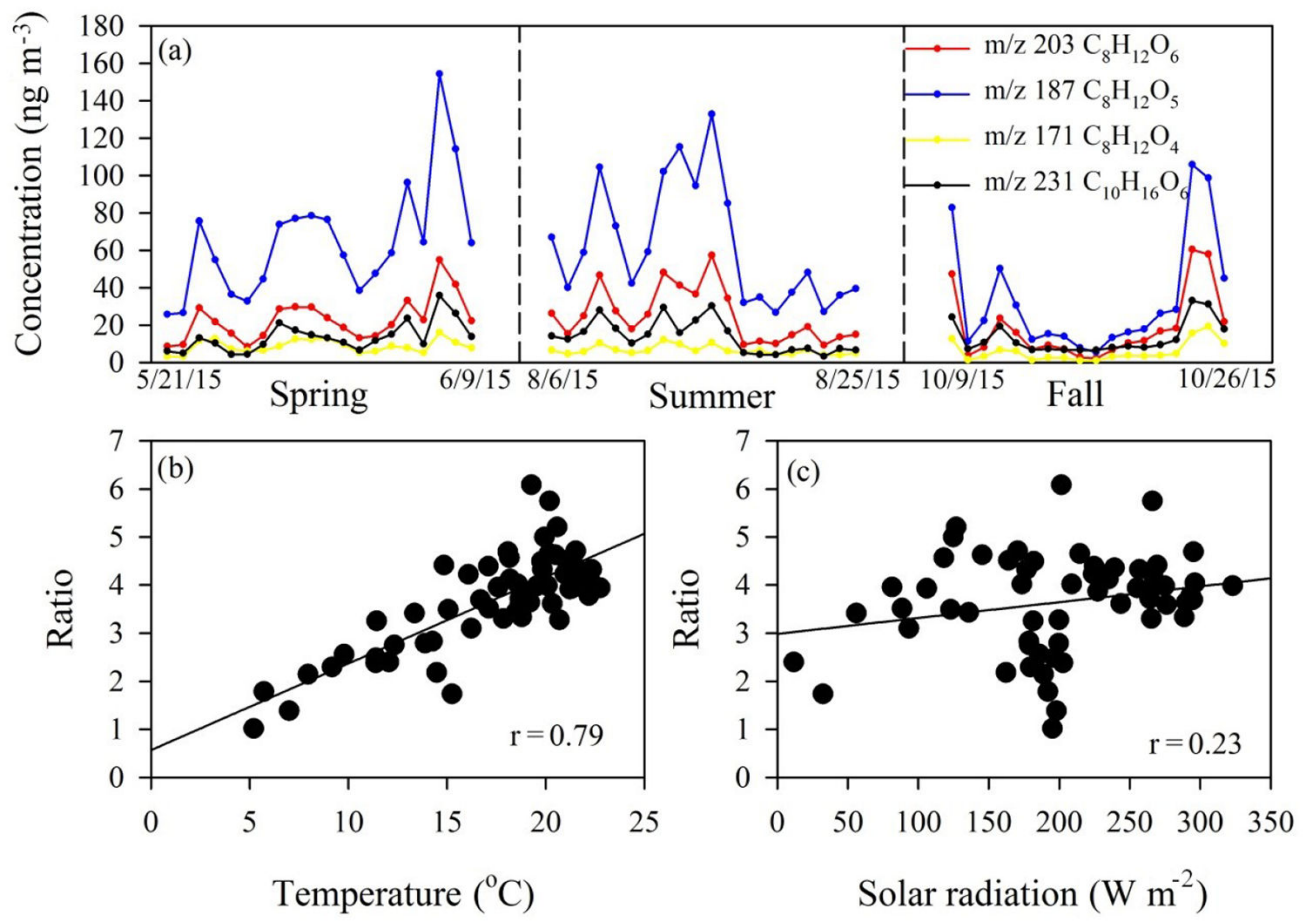
**(a)** Time series of these four identified terpenoic acids: 3-methyl-1,2,3-butanetricarboxylic acid (MBTCA, C_8_H_12_O_6_ *m/z* 203), 2-hydroxyterpenylic acid (C_8_H_12_O_5_, *m/z* 187), terpenylic acid (C_8_H_12_O_4_, *m/z* 171), and diaterpenylic acid acetate (DTAA, C_10_H_16_O_6_, *m/z* 231); **(b)** correlation of concentration ratios of higher-generation oxidation products (C_8_H_12_O_6_, *m/z* 203 and C_8_H_12_O_5_, *m/z* 187) to early oxidation fresh SOA products (C_8_H_12_O_4_, *m/z* 171 and C_10_H_16_O_6_, *m/z* 231) with temperature and **(c)** with solar radiation.

Concentrations of nitro-aromatics, organosulfate–nitrooxy-organosulfate, and terpenoic acids are summarized in Tables 3, S2, S3, and S4. A time series of compound class totals are presented in Fig. 2. Generally negligible concentrations of nitro-aromatics were observed during spring and summer except for occasional spikes. However, higher concentrations of nitro-aromatics were observed in the fall when moderate correlations were observed with levoglucosan (Fig. 3, *r* ≥ 0.5, *p* < 0.01; see Table S6 for correlation coefficients). A residential wood burning contribution is likely given the lower temperatures observed during this season. Similar positive correlations between nitro-aromatics and wood burning are also reported during the winter season (Gaston et al., 2016; Kahnt et al., 2013; Kitanovski et al., 2012; Iinuma et al., 2010, 2016). Smoke at the sampling site on 19 and 21 October coincided with firewood burning at the main office of the Coweeta Hydrologic Laboratory immediately adjacent to the sampling location. Nitro-aromatics were relatively elevated, but no significant increase in organosulfates or terpenoic acids was found from these fresh smoke events. In contrast, an example of an aged biomass burning signal is illustrated on 24 and 25 October. Pronounced spikes of nitrocatechol (C_6_H_5_NO_4_), methyl-nitrocatechol (C_7_H_7_NO_4_), and levoglucosan were observed (Fig. 3), along with elevated concentrations of organosulfates, OC, and aged biogenic aerosol tracers (terpenoic acids *m/z* 203 and 187 shown in Fig. 4a; a detailed discussion can be found in the subsequent section). However, EC was only slightly higher. This event did not correspond to local burning at Coweeta and was most likely associated with long-range transport. Clustering of backward trajectories (120 h duration for individual trajectories; 48 total trajectories covering the 2-day event) suggests that northeast Georgia (shown in Fig. S5 in the Supplement) is the most likely origin of the biomass burning event observed on 24 and 25 October.

Nitro-aromatics correlated with EC across the seasons; both were likely emitted from biomass burning (Gaston et al., 2016; Iinuma et al., 2010; Kahnt et al., 2013; Mohr et al., 2013). Interestingly, light absorption at *λ* = 365 nm was highly correlated (*r* = 0.80, *p* < 0.01) with nitro-aromatics in the fall when nitro-aromatic concentrations were elevated. In addition, NO_*x*_ correlated inversely (*r* = −0.72, *p* < 0.01) with temperature in the fall. Lower fall temperatures in the region may have resulted in frequent residential wood burning, which emits NO_*x*_ and light-absorbing BrC (e.g., nitro-aromatics; Liu et al., 2015; Mohr et al., 2013). Although nitro-aromatics account for a minor fraction of OM, they could potentially contribute to 4% of light absorption by BrC (Mohr et al., 2013). Overall, nitro-aromatics displayed relatively week correlation with WSON (*r* < 0.65) across all seasons; the extremely low concentrations observed suggest a generally small contribution of nitro-aromatics to WSON at the sampling site; hence the lack of strong correlation.

### 3.4 Organosulfates and nitrooxy-organosulfates

Organosulfate concentrations were highest in summer and lowest in fall (Table 3), contributing 3.9 and 1.0% *w/w* of organic matter (OM; estimated by applying an OM/OC factor of 2) mass, respectively, during these seasons. Organosulfate formation is an example of heterogeneous chemistry involving the uptake of reactive precursors on acidified sulfate aerosols requiring a mixture of biogenic and anthropogenic emissions. The air masses at Coweeta are mainly from the southwest and westerly directions in spring and summer, but during fall may become more stagnant and slow moving during southwesterly conditions or shift to the northwest (see clustered back trajectories shown in Fig. S4). Because Atlanta, GA is southwest of Coweeta, southwesterly flow during spring and summer may be associated with the long-range transport of urban pollutants and precursors, including sulfate and sulfuric acid, leading to elevated organosulfate formation compared to fall when the prevailing wind direction changes.

Among all organosulfates identified, the isoprene-derived organosulfate (*m/z* 215, 2-methyltetrol derived), which is formed from isoprene-derived epoxydiols (IEPOX) under low NO_*x*_ conditions, was the most abundant; concentrations reached 167 ng m^−3^ in summer. Similar high concentrations were also reported in ambient samples collected at other sites in the southeastern US (Lin et al., 2013b; Worton et al., 2013). Of the six nitrooxy-organosulfates identified, isoprene-derived *m/z* 260 was most abundant, approximately 6-fold higher than monoterpene-derived *m/z* 294 nitrooxy-organosulfate.

A subset of possible organosulfates and nitrooxy-organosulfates produced from isoprene and monoterpene oxidation exhibited strong correlations with distinctive SOA tracers (e.g., markers 2-methylglyceric acid, C-5 alkene triols and methyltetrols for isoprene oxidation products; tracer 3-hydroxyglutaric acid for pinene oxidation products; see Table S7). Lack of correlation between nitrooxy-organosulfate *m/z* 294 and 3-hydroxyglutaric acid may indicate a favored nighttime nitrate radical formation pathway over photochemical oxidation. Given that NO_*x*_ levels at the rural Coweeta sampling site were typically less than 1 ppb, photooxidation pathways involving high [NO_*x*_] to form nitrooxy-organosulfates are less likely. Though a contribution from photochemical oxidation cannot be ruled out (Lee et al., 2016; Romer et al., 2016), nighttime nitrate radical chemistry is most likely the dominating formation mechanism under such conditions. In contrast to our observations, He et al. (2014) reported good correlations (*r* > 0.5, *p* < 0.01) of *m/z* 294 with 3-hydroxyglutaric acid and higher daytime *m/z* 294 concentrations for summer samples collected in Pearl River Delta, China, where a seasonal average NO_*x*_ level of 30 ppb was observed. The authors suggested that the dominant *m/z* 294 formation pathway was through daytime photochemistry rather than nighttime NO_3_ chemistry. The extremely low NO_*x*_ levels at our study site compared to that measured by He et al. (2014) may explain the opposite behavior in terms of *m/z* 294 formation mechanisms.

Organosulfates exhibited statistically significant correlations with WSON only in the summer (*r* = 0.64, *p* < 0.01), which reflected the importance of N-containing organosulfates or their formation chemistry to WSON during summer compared to the other seasons. During this season, nitrooxy-organosulfates accounted for ~ 2% of bulk WSON on average. A strong correlation may therefore not be expected.

### 3.5 Terpenoic acids

Terpenoic acids, which provide insight into the extent of biogenic compound oxidation and atmospheric processing (i.e., aerosol aging), were the most abundant group of compounds relative to nitro-aromatics and organosulfates. On average, terpenoic acids accounted for 6.5 to 8.7% *w/w* of OM in PM_2.5_. The warmer spring and summer periods show higher production of terpenoic acids compared to the cool and drier fall season. Higher emissions of biogenic VOC precursors and higher solar radiation intensities during warm seasons, which drive photochemistry, are factors contributing to observed seasonal variability.

The terpenoic acids correlated well with WSOC and OC (Table 2). This is expected as terpenoic acids accounted for a substantial portion of OM at the site. Individual acids (except compounds C_7_H_10_O_4_ and C_9_H_14_O_4_) exhibited strong correlations with the pinene-derived SOA tracer 3-hydroxyglutaric acid (*r* > 0.75, *p* < 0.01; correlation coefficients shown in Table S8), indicating the presence of *α*- and *β*-pinene oxidation products. The poor correlations between acids C_7_H_10_O_4_ (*m/z* 157) and C_9_H_14_O_4_ (*m/z* 185) suggest the presence of biogenic VOC precursors other than *α*- and *β*-pinene, such as limonene and Δ^3^-carene (Gomez-Gonzalez et al., 2012).

Recent chamber studies identified several terpenoic acid structures that were also observed in ambient aerosol samples, including 3-methyl-1,2,3-butanetricarboxylic acid (MBTCA, C_8_H_12_O_6_, *m/z* 203), 2-hydroxyterpenylic acid (C_8_H_12_O_5_, *m/z* 187), terpenylic acid (C_8_H_12_O_4_, *m/z* 171), and diaterpenylic acid acetate (DTAA, C_10_H_16_O_6_, *m/z* 231; Claeys et al., 2009; Kahnt et al., 2014). MBTCA and 2-hydroxyterpenylic acid have been identified as highly oxygenated, higher-generation *α*-pinene SOA markers and observed in high abundance in ambient aerosols (Gomez-Gonzalez et al., 2012; Kahnt et al., 2014; Müller et al., 2012; Szmigielski et al., 2007). Additionally, terpenylic acid and DTAA are characterized as early photooxidation products from *α*-pinene ozonolysis. Claeys et al. (2009) proposed further oxidation processes (aging) of terpenylic acid involving OH radical chemistry to form 2-hydroxyterpenylic acid. Figure 4 provides a time series of the terpenoic acids identified in this study. In general, 2-hydroxyterpenylic acid was the most abundant species across the seasons. To assess the extent of aging, concentration ratios of higher-generation oxidation products (C_8_H_12_O_6_, *m/z* 203 and C_8_H_12_O_5_, *m/z* 187) to early oxidation fresh SOA products (C_8_H_12_O_4_, *m/z* 171 and C_10_H_16_O_6_, *m/z* 231) are calculated. Estimated seasonal averages of these ratios are 3.98, 4.37, and 2.44 for spring, summer, and fall, respectively. Thus, during spring and summer, aerosols observed at the site were more aged. Figure 4 shows the correlation of these ratios with temperature (*r* = 0.79, *p* < 0.001) and solar radiation (*r* = 0.23, *p* < 0.1). A clear relationship between temperature and OH-radical-initiated oxidation (aging) is evident. However, oxidation appears less dependent on solar radiation at our sampling site. A similar higher contribution of these aged biogenic SOA tracers was also reported under warm summer conditions characterized by high temperature and high solar radiation (Claeys et al., 2012; Gomez-Gonzalez et al., 2012; Hamilton et al., 2013; Kahnt et al., 2014). Based on the typical chemical lifetime of biogenic SOA by OH oxidation and the precipitation frequency at the Coweeta site, biogenic SOA collected at Coweeta probably had an atmospheric lifetime of several days before depletion by oxidation processes and/or scavenging by precipitation (Epstein et al., 2014).

Terpenoic acids may also provide some insight into the formation mechanisms of organosulfates. While organosulfate concentrations were highest during summer, correlations with ${\text{SO}}_{4}^{2-}$ were strongest during spring and fall and weakest during summer. Conversely, organosulfates and terpenoic acids correlated strongly (*r* = 0.91. *p* < 0.01) during summer. Terpenoic acids are either first- or second-generation oxidation products from gas-phase monoterpenes; particulate ${\text{SO}}_{4}^{2-}$ abundance should not substantially influence the gas–particle partitioning of terpenoic acids. The strong correlation between organosulfates and terpenoic acids in summer suggests that organosulfate formation is limited by monoterpene emissions rather than ${\text{SO}}_{4}^{2-}$ availability, while in the spring and fall (especially fall), organosulfate production may be more limited by ${\text{SO}}_{4}^{2-}$. The degree of particle neutralization, calculated as the molar ratio of ${\text{NH}}_{4}^{+}$ to the sum of ${\text{SO}}_{4}^{2-}$ and ${\text{NO}}_{3}^{-}$, averaged 0.94, 0.98, and 0.94 for spring, summer, and fall, respectively. Neutralization being close to but less than unity implies that aerosols are slightly acidic at the site. Chamber studies have illustrated that acidified ${\text{SO}}_{4}^{2-}$ could enhance heterogeneous reactions to form SOA from isoprene and monoterpenes (Iinuma et al., 2009; Surratt et al., 2007, 2010). Similar positive correlations observed at the Coweeta site were also found between isoprene tracers, including isoprene-derived organosulfates and ${\text{SO}}_{4}^{2-}$, by Lin et al. (2013b) at a rural site in the southeastern US. However, in contrast to chamber experiments, this study and other ambient field measurements have not provided clear evidence of the acidity enhancement of organosulfate formation (He et al., 2014; Lin et al., 2013b; Worton et al., 2011), indicating possible differences in exact mechanisms and processing to form these organosulfates under atmospheric conditions relative to chamber studies. Recent mechanistic modeling simulations by Budisulistiorini et al. (2017) suggest that the role of sulfate in IEPOX–organosulfate formation might be through the surface area uptake of IEPOX and the rate of particle-phase reaction.

Very good correlations between WSON and terpenoic acids were observed during summer and fall (*r* ≥ 0.7, *p* < 0.01). Given the secondary nature of terpenoic acids, this finding may suggest that WSON during these two seasons is associated with more aged air masses and perhaps dominated by secondary organic components rather than primary emitted N-containing constituents such as pollens, fungi, and bacteria (Elbert et al., 2007; Miyazaki et al., 2014).

The contribution of identified N-containing species to WSTN and WSON nitro-aromatics and nitrooxy-organosulfates combined were estimated to account for as much as 28% of WSON for samples impacted by local biomass burning, which reflected the abundance and potential importance of these groups of species to the atmospheric N-deposition budget. Seasonal average ratios of identified WSON to WSTN ranged from 1.0 to 4.4% with the highest recorded for fall (Table 4). Nitrooxy-organosulfates dominated over nitro-aromatics as a source of organic nitrogen, contributing > 90% to identified WSON across seasons. However, during episodes of biomass burning, nitro-aromatics contributed as much as 32% of identified WSON compounds. The ratio of WSON to WSOC was estimated to be 0.05, 0.04, and 0.02 for spring, summer, and fall, which implies that organic N is most enriched during spring, reflecting a spring maximum in seasonal emissions of organic N from biological sources (e.g. pollens, spores, leaf litter decomposition) combined with smaller contributions from secondary atmospheric processes. The observed WSON/WSOC ratios in this study were slightly lower than those reported for other forest sites (0.03–0.09; Lin et al., 2010; Miyazaki et al., 2014), which are not as remote and pristine as the forest site in this study. Anthropogenic influences at the study sites described by Lin et al. (2010) and Miyazaki et al. (2014), such as [${\text{SO}}_{4}^{2-}$] and [NO_*x*_], were ~ 5 times higher than those measured at the Coweeta site. Concentration-weighted average WSON/WSOC ratios for identified compounds (nitro-aromatics, organosulfates–nitrooxy-organosulfates, and terpenoic acids) in this study were estimated to be 0.003. This value is 10 times less than the overall WSON/WSOC ratio observed at the site, which indicates the existence of other higher-N-content species in the aerosols. Moreover, the identified ON/WSON percentage was estimated to be 1.0, 2.0, and 4.4 for spring, summer, and fall, respectively. Such differences further suggest that much more unidentified WSON compounds exist in spring when organic N is most enriched from biological processes.

### 3.6 PMF analysis

**Figure 5.**
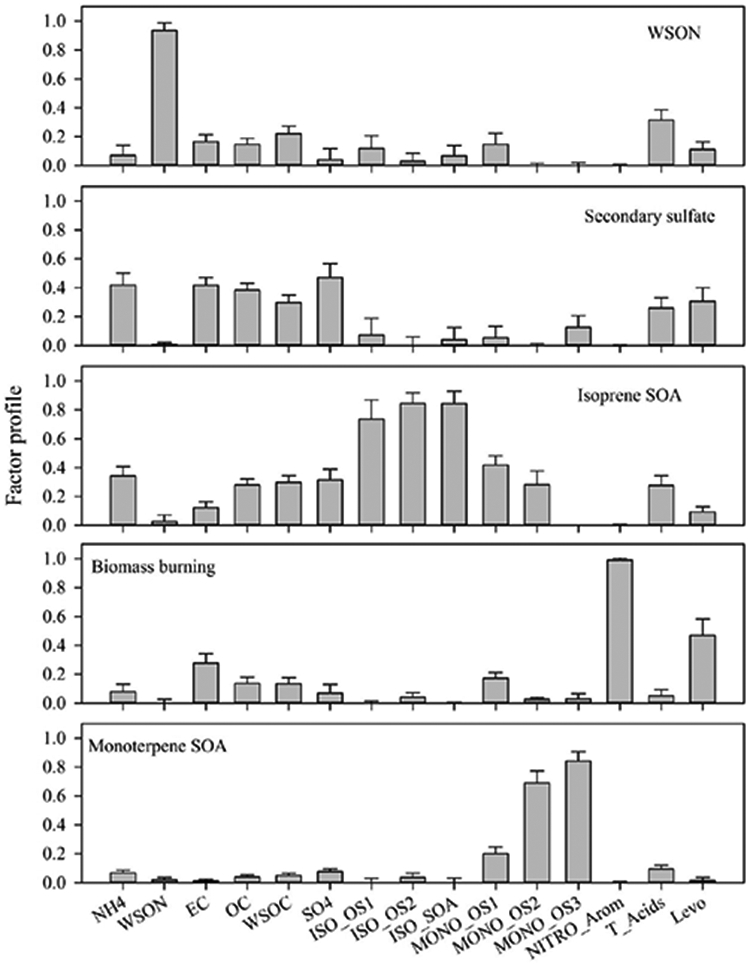
Normalized factor profiles (error bar represents 1 standard deviation).

**Figure 6.**
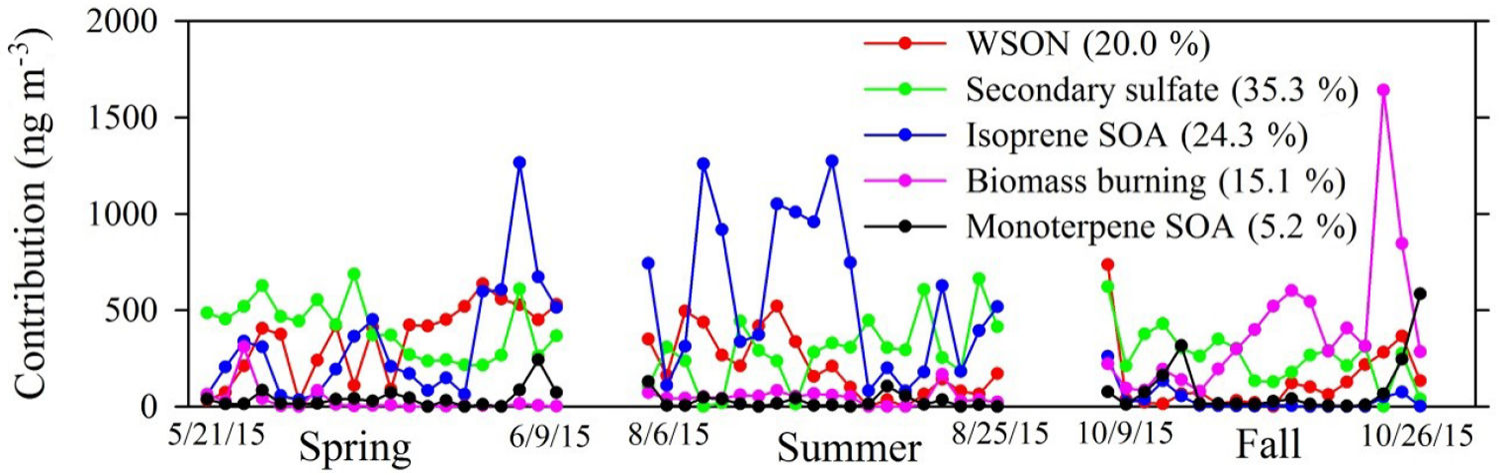
Time series of factor contributions to WSOC (mean factor contribution shown in brackets).

PMF analysis was conducted to identify individual source contributions to total WSOC. Factor profiles and time series of factor contributions are presented in Figs. 5 and 6. Listed in order of percent contribution to WSOC, the five factors that were resolved include secondary sulfate processing (35.3%), isoprene SOA (24.3%), WSON-containing OM (20.0%), biomass burning (15.1%), and monoterpene SOA (5.2%). Overall, these factors could explain 89 ± 2% of observed WSOC (*r* = 0.88, *p* < 0.0001). The secondary sulfate profile contained a signature of high ${\text{SO}}_{4}^{2-}$, which was most likely present as fine particulate (NH_4_)_2_ SO_4_ and NH_4_HSO_4_. Secondary sulfate was the most important factor during spring, though it was a significant contributor in summer and fall as well. Isoprene SOA, which was identified based on isoprene-derived organosulfates and isoprene SOA markers, was the most important factor during summer. The biomass burning factor, which exhibited a high portion of nitro-aromatic and levoglucosan markers, dominated in the fall. This pattern agreed well with observed patterns of nitro-aromatic compounds. Monoterpene SOA, which was resolved based on the composition of monoterpene-derived organosulfates, was overall a minor contributor with the exception of a few samples during the fall intensive.

WSON-containing OM contributed 20% to WSOC overall, demonstrating a significant association between organic N and C in PM_2.5_ at our study site. The WSON-containing OM source profile exhibited weak correlation with most measured species with the exception of modest correlations with terpenoic acids. WSON-containing OM contributed more to WSOC in late spring and early summer, which was consistent with the observed higher production of nitrooxy-organosulfates during these sampling periods as well as terpenoic acids. The relationship with terpenoic acids may reflect an association of WSON with more aged air masses. Because nitro-aromatics and nitrooxy-organosulfates contribute only a small portion of WSON on average, the 20% contribution of WSON-containing OM to WSOC primarily reflects the contribution of organic N present in bulk WSON but unspeciated in this work.

## 4 Conclusions

Ambient PM_2.5_ collected at a temperate mountainous forest site was investigated on a bulk chemical and a molecular level during spring, summer, and fall of 2015. Analyses focused on the speciation of nitro-aromatics associated with biomass burning, organosulfates produced from biogenic SOA precursors, and terpenoic acids formed from monoterpene oxidation. Among these three groups, terpenoic acids were estimated to be most abundant, contributing up to a seasonal average of 8.7% of OM in PM_2.5_ during spring. Warm periods in spring and summer exhibited the highest production of terpenoic acids, when SOA correspondingly showed a higher degree of aging. The relative abundance of aged biogenic SOA tracers (MBTCA and 2-hydroxyterpenylic acid), which reflect the degree of organic aerosol aging, showed a strong correlation with temperature. Such a relationship might indicate temperature dependence of OH-radical-initiated oxidation steps or aging in the formation of higher-generation oxidation products.

Organosulfates showed a peak in summer and lowest concentrations during fall, contributing averages of 3.9 and 1.0% of OM mass, respectively, during these seasons. Isoprene-derived organosulfate (*m/z* 215, 2-methyltetrol derived), formed from isoprene-derived epoxydiols (IEPOX) under low NO_*x*_ conditions, was the most abundant identified organosulfate (up to 167 ng m^−3^ in summer). This observation is consistent with observations of low NO_*x*_ levels (< 1 ppb on average) at our study site. Nighttime nitrate radical chemistry is most likely the dominant formation mechanism for nitrooxy-organosulfates measured at this remote site with background-level NO_*x*_.

Nitro-aromatics were most abundant at our study site during the fall (up to 0.01% of OM mass). Moderate correlations were observed between nitro-aromatics and the biomass burning marker levoglucosan, indicating a common origin. Nitro-aromatics also correlated well with EC across seasons. The highest concentrations of nitro-aromatics, specifically nitrocatechol and methyl-nitrocatechol, were associated with aged biomass burning plumes as indicated by correspondingly high concentrations of terpenoic acids.

Bulk measurements determined that WSOC accounted for 62 ± 13% of OC throughout the entire study period without significant seasonal variability. PMF analysis indicated that a significant portion of this organic carbon was associated with a resolved factor of WSON-containing OM. As a component of total nitrogen in PM_2.5_, the largest contributions of WSON to WSTN were observed in spring (~ 18% *w/w*) and the lowest in the fall (~ 10% *w/w*). On average, identified nitro-aromatic and nitrooxy-organosulfate compounds accounted for a small fraction of WSON, ranging from ~ 1% in spring to ~ 4% in fall, though they were observed to contribute as much as 28% *w/w* of WSON in individual samples that were impacted by local biomass burning. Of the organic N compounds speciated in this study, nitrooxy-organosulfates dominated over nitro-aromatics as a source of organic nitrogen, contributing > 90% to WSON across seasons. As a component of WSON, nitro-aromatics were most important during episodes of biomass burning, when their contribution to identified and total WSON was as much as 32% and 3%, respectively. Concentration-weighted average WSON/WSOC ratios for compounds identified in this study were estimated to be 0.003. This number is an order of magnitude lower than the overall WSON/WSOC ratio observed, indicating a predominance of other uncharacterized N species. Other N-containing substituents of WSON could include amino acids, amines, urea, and N-heterocyclic compounds, as well as substances of biological origin such as spores, pollens, and bacteria (Cape et al., 2011; Neff et al., 2002a). Ratios of WSON to WSOC indicate that organic C is most enriched by organic N during spring, perhaps reflecting a spring maximum in seasonal emissions of organic N from biological sources combined with smaller contributions from secondary atmospheric processes (e.g., nitrooxy-organosulfates).

Although nitro-aromatics and nitrooxy-organosulfates contribute a relatively small fraction of organic N in PM_2.5_ at our study site, our observations shed light on this complex but largely unknown portion of the atmospheric N budget. Our results provide further understanding of the patterns and composition of SOA in a remote mountain environment previously uncharacterized. Similar to our results, other studies generally find that individual groups of organic N compounds (e.g., amines, amino acids, urea) cannot explain the majority of bulk WSON (Cape et al., 2011; Day et al., 2010; Place et al., 2017; Samy et al., 2013), which globally accounts for ~ 25% of total N in rainfall (Cape et al., 2011; Jickells et al., 2013). As methodological advances allow for greater speciation of this large pool of atmospheric N, future work should emphasize the analysis of both primary and secondary forms of organic N in individual samples, in addition to bulk analyses, so that a more complete picture of organic N composition may be developed for specific atmospheric chemical and meteorological conditions. Additionally, as progress is made in better characterizing the composition and sources of atmospheric organic N, the ecological and atmospheric science communities must work together to develop a better understanding of the role of atmospheric organic N in ecosystem N cycling.

## Supplementary Material

Sup1

## Figures and Tables

**Table 1.** Summary of particulate and gaseous species measured at the Coweeta sampling site in 2015.

(μg m^−3^	Spring				Summer				Fall			
	Mean	Median	Min	Max	Mean	Median	Min	Max	Mean	Median	Min	Max
OM (OC · 2)	3.77	3.41	2.18	7.52	3.80	3.79	2.00	6.32	3.36	2.85	1.96	7.49
EC	0.05	0.05	0.03	0.10	0.05	0.05	0.02	0.08	0.07	0.07	0.03	3.75
WSOC	1.14	1.03	0.45	2.47	1.22	1.24	0.53	2.34	1.09	0.78	0.50	3.25
WSTN	0.33	0.29	0.14	0.86	0.34	0.32	0.11	0.59	0.21	0.20	0.08	0.52
WSON	0.06	0.07	ND	0.14	0.05	0.03	ND	0.11	0.03	0.02	ND	0.13
${\text{NH}}_{4}^{+}\text{-N}$	0.27	0.24	0.08	0.74	0.29	0.28	0.09	0.48	0.18	0.17	0.08	0.38
${\text{NO}}_{3}^{-}\text{-N}$	0.00	0.00	ND	0.01	0.00	0.00	ND	0.01	0.00	0.00	ND	0.01
${\text{NO}}_{2}^{-}\text{-N}$	0.00	0.00	ND	0.00	0.00	0.00	ND	0.01	0.00	0.00	ND	0.00
${\text{SO}}_{4}^{2-}$	0.99	0.93	0.26	2.44	1.01	0.95	0.31	1.85	0.63	0.58	0.30	1.33
O_3_ (ppb)	25.1	21.6	13.9	46.1	15.8	15.8	9.0	22.8	19.4	20.5	11.1	26.9
NO_*x*_ (ppb)	0.75	0.79	0.45	1.03	0.54	0.58	0.24	0.91	0.65	0.68	0.43	0.89
Temp (°C)	18.4	18.6	14.8	22.3	20.7	20.6	18.1	22.8	11.6	11.7	5.2	17.1
RH%	81.7	84.9	61.0	94.8	82.1	83.1	71.9	88.5	77.7	74.9	65.1	92.0
Radiation (W m^−2^)	235	265	81	296	205	201	106	323	151	180	12	203

**Table 2.** Spearman rank correlation coefficients among measured species and meteorological variables by season. Nitro-aromatics (Nitro), Organosulfates (OS), and terpenoic acids (Tacids) represent group summed concentrations.

Spring	OC	WSOC	${\text{NO}}_{3}^{-}$	${\text{NH}}_{4}^{+}$	${\text{SO}}_{4}^{2-}$	WSON	Abs_365_	Nitro	OS	Tacids	O_3_	NO_*x*_	Temp	RH	radiation	Precip
EC	**0.853**	0.474	0.177	**0.690**	**0.705**	0.129	**0.875**	**0.583**	**0.645**	**0.579**	0.430	0.263	0.364	**−0.627**	0.520	−0.458
OC		**0.737**	0.069	**0.767**	**0.708**	0.328	**0.773**	0.541	**0.848**	**0.761**	0.275	0.498	0.543	−0.408	0.441	−0.315
WSOC			0.105	0.523	0.429	**0.768**	0.424	0.241	**0.805**	**0.723**	0.185	0.543	0.472	−0.059	0.135	−0.145
${\text{NO}}_{3}^{-}$				0.15	0.137	0.129	0.108	0.492	−0.104	−0.051	0.559	0.084	−0.203	**−0.564**	0.362	−0.169
${\text{NH}}_{4}^{+}$					**0.944**	0.457	**0.842**	0.355	**0.684**	0.298	0.474	0.189	0.439	−0.510	0.441	−0.362
${\text{SO}}_{4}^{2-}$						0.400	**0.827**	0.277	**0.642**	0.229	0.457	0.051	0.540	−0.526	0.374	−0.306
WSON							0.215	−0.113	0.522	0.236	0.215	0.140	0.326	0.080	−0.105	0.055
Abs_365_								0.456	**0.591**	0.349	0.495	0.174	0.254	**−0.612**	0.507	−0.529
Nitro^1^									0.278	0.426	0.493	0.319	0.021	−0.537	0.307	−0.177
OS^2^										**0.759**	0.080	0.341	**0.644**	−0.084	0.162	−0.140
Tacids^3^											−0.066	**0.571**	0.442	0.000	0.141	−0.066
O_3_												0.068	0.026	**−0.797**	0.453	−0.219
NO_*x*_													0.227	−0.068	0.257	−0.165
Temp														−0.111	0.183	0.061
RH															**−0.786**	0.551
Radiation																**−0.734**
Summer	OC	WSOC	${\text{NO}}_{3}^{-}$	${\text{NH}}_{4}^{+}$	${\text{SO}}_{4}^{2-}$	WSON	Abs_365_	Nitro	OS	Tacids	O_3_	NO_*x*_	Temp	RH	radiation	Precip
EC	**0.671**	**0.659**	0.113	**0.626**	0.555	**0.562**	0.546	**0.576**	0.474	0.537	0.325	0.242	−0.402	−0.384	0.465	−0.356
OC		**0.961**	0.233	**0.627**	0.517	0.556	0.558	0.523	**0.856**	**0.823**	0.304	0.289	−0.379	−0.300	0.269	−0.189
WSOC			0.263	**0.592**	0.490	0.549	0.397	**0.564**	**0.820**	**0.835**	0.247	0.238	−0.302	−0.325	0.259	−0.269
${\text{NO}}_{3}^{-}$				0.343	0.271	0.355	−0.143	0.165	0.325	0.469	**0.642**	**0.665**	0.120	−0.279	0.263	0.181
${\text{NH}}_{4}^{+}$					**0.977**	0.550	0.405	0.535	**0.609**	**0.585**	0.320	0.415	−0.108	−0.388	0.421	−0.218
${\text{SO}}_{4}^{2-}$						0.465	0.343	0.477	0.487	0.474	0.241	0.350	−0.090	−0.426	0.447	−0.290
WSON							0.170	**0.633**	**0.642**	**0.692**	**0.698**	0.391	0.026	**−0.637**	0.555	−0.201
Abs_365_								0.086	0.423	0.278	0.149	0.140	**−0.586**	0.012	0.167	0.089
Nitro									**0.573**	**0.614**	0.367	0.418	−0.116	−0.346	0.247	−0.446
OS										**0.905**	0.338	0.472	−0.080	−0.175	0.098	0.087
Tacids											0.432	0.531	−0.150	−0.263	0.138	−0.035
O_3_												**0.621**	−0.045	**−0.607**	**0.571**	−0.046
NO_*x*_													−0.116	−0.049	0.018	0.214
Temp														−0.097	−0.012	0.172
RH															**−0.919**	**0.607**
Radiation																**−0.583**
Fall	OC	WSOC	${\text{NO}}_{3}^{-}$	${\text{NH}}_{4}^{+}$	${\text{SO}}_{4}^{2-}$	WSON	Abs_365_	Nitro	OS	Tacids	O_3_	NO_*x*_	Temp	RH	radiation	Precip
EC	**0.719**	**0.695**	0.449	0.216	0.127	**0.707**	**0.897**	**0.779**	0.154	0.472	0.042	0.106	−0.036	−0.044	−0.100	−0.380
OC		**0.955**	0.077	0.434	0.333	**0.837**	**0.715**	0.340	0.554	**0.897**	−0.282	−0.189	0.525	0.441	−0.441	0.047
WSOC			0.092	**0.593**	0.494	**0.816**	**0.668**	**0.362**	**0.649**	**0.922**	−0.222	−0.152	0.474	0.422	−0.470	0.146
${\text{NO}}_{3}^{-}$				−0.044	−0.053	0.106	0.385	0.445	−0.300	−0.088	0.257	0.084	−0.375	−0.461	0.265	−0.385
${\text{NH}}_{4}^{+}$					**0.983**	0.490	0.191	0.209	**0.874**	**0.664**	−0.158	−0.096	0.356	0.350	−0.410	0.265
${\text{SO}}_{4}^{2-}$						0.399	0.100	0.152	**0.833**	0.571	−0.110	−0.086	0.313	0.290	−0.342	0.244
WSON							**0.789**	0.486	0.546	**0.746**	−0.143	0.036	0.364	0.441	−0.538	0.224
Abs_365_								**0.802**	0.110	0.494	0.150	0.286	−0.096	0.011	−0.226	−0.273
Nitro									0.001	0.187	0.313	0.445	−0.455	−0.226	0.009	−0.378
OS										**0.746**	−0.350	−0.356	**0.659**	0.573	−0.581	0.466
Tacids											−0.401	−0.249	**0.653**	**0.628**	−0.587	0.241
O_3_												**0.664**	**−0.746**	**−0.820**	**0.602**	−0.340
NO_*x*_													**−0.719**	−0.418	0.389	−0.303
Temp														**0.787**	**−0.639**	0.490
RH															**−0.847**	**0.638**
Radiation																**−0.640**
Overall	OC	WSOC	${\text{NO}}_{3}^{-}$	${\text{NH}}_{4}^{+}$	${\text{SO}}_{4}^{2-}$	WSON	Abs_365_	Nitro	OS	Tacids	O_3_	NO_*x*_	Temp	RH	radiation	Precip
EC	**0.545**	**0.422**	**0.361**	0.214	0.216	0.175	**0.753**	**0.642**	−0.041	0.283	0.338	0.308	**−0.396**	**−0.500**	0.131	**−0.449**
OC		**0.928**	0.087	**0.672**	**0.611**	**0.585**	**0.615**	0.181	**0.698**	**0.828**	0.016	0.167	0.281	−0.017	0.207	−0.115
WSOC			0.110	**0.643**	**0.564**	**0.726**	**0.444**	0.120	**0.729**	**0.848**	−0.025	0.116	0.325	0.102	0.172	−0.054
${\text{NO}}_{3}^{-}$				0.002	0.018	0.161	0.200	0.310	−0.190	0.097	**0.536**	**0.384**	−0.322	**−0.433**	0.189	−0.127
${\text{NH}}_{4}^{+}$					**0.976**	**0.543**	**0.358**	−0.063	**0.794**	**0.567**	0.071	0.053	**0.528**	−0.061	0.313	−0.061
${\text{SO}}_{4}^{2-}$						**0.493**	0.348	−0.103	**0.733**	**0.502**	0.107	0.029	**0.517**	−0.107	**0.339**	−0.072
WSON							0.272	−0.061	**0.575**	**0.590**	0.233	0.185	**0.356**	0.103	0.283	0.044
Abs_365_								**0.372**	0.127	0.294	0.303	0.260	−0.218	−0.290	0.100	−0.255
Nitro									−0.302	0.004	0.245	0.153	**−0.570**	**−0.467**	−0.177	−0.332
OS										**0.721**	−0.138	0.004	**0.742**	0.234	0.244	0.188
Tacids											−0.031	0.230	**0.352**	0.295	0.086	0.131
O_3_												**0.572**	−0.283	**−0.574**	**0.482**	−0.152
NO_*x*_													−0.200	−0.086	0.287	−0.035
Temp														0.272	0.238	0.242
RH															**−0.498**	**0.618**
Radiation																**−0.492**

1 Nitro-aromatics

2 organosulfates

3 terpenoic acids; values in bold indicate *p* < 0.01.

**Table 3.** Seasonal statistics of measured groups of compounds.

(ng m^−3^)	Spring				Summer				Fall			
	Mean	Median	Min	Max	Mean	Median	Min	Max	Mean	Median	Min	Max
Nitro-aromatics	0.07	0.00	ND	0.81	0.02	0.02	ND	0.04	0.28	0.17	0.04	1.78
Organosulfates^1^	96.77	83.05	33.07	255.17	153.36	125.41	38.93	306.66	34.69	15.27	0.17	118.68
Terpenoic acids	325.62	304.05	128.68	771.16	294.01	249.19	115.08	634.99	250.66	148.91	52.94	809.46
% of OM^2^												
% nitro-aromatics	0.00	0.00	ND	0.02	0.00	0.00	ND	0.00	0.01	0.01	0.00	0.02
% organosulfates	2.47	2.42	1.19	3.64	3.87	3.80	1.95	5.56	0.98	0.63	0.31	2.21
% terpenoic acids	8.65	8.29	4.62	12.88	7.50	7.77	3.80	11.64	6.48	5.21	2.70	12.00

1 Including nitrooxy-organosulfates

2 percent contribution of each group of identified compounds (combined total) to organic matter.

**Table 4.** Ratios of identified nitrogen-containing compounds (nitro-aromatics and nitrooxy-organosulfates) to WSON.

(ng N m^−3^)	Spring				Summer				Fall			
	Mean	Median	Min	Max	Mean	Median	Min	Max	Mean	Median	Min	Max
WSON	59	74	ND	140	46	33	ND	105	25	15	ND	133
Identified ON	0.48	0.36	0.1	1.75	0.65	0.53	0.12	1.83	0.46	0.26	0.07	1.70
Identified ON/WSON %	1.02	0.64	ND	3.09	2.04	1.71	ND	7.84	4.37	1.50	ND	27.90
