# Supplementary material for: Characterization of organic nitrogen in aerosols at a forest site in the southern Appalachian Mountains
Source: Atmos Chem Phys. Author manuscript; Available in PMC 2020 Jul 23. (PMC7377252; doi:10.5194/acp-18-6829-2018)
Supplement: Sup1 [file NIHMS1540579-supplement-Sup1.pdf]

*Supplement of*

# Characterization of organic nitrogen in aerosols at a forest site in the southern Appalachian Mountains

**Xi Chen et al.**

*Correspondence to:* John T. Walker (walker.johnt@epa.gov)

Table S1. List of quantified target compounds by GCMS with surrogate compound and quantitation ions.

| | Quantified as | Characterized ions | Mean Concentration (Range) ($ng/m^3$) |
|---|---|---|---|
| *Isoprene SOA markers* | | | |
| 2-Methylglyceric acid | meso-erythritol | 219, 233, 306, 321 | 1.55(0.20-4.79) |
| 2-Methylthreitol | meso-erythritol | 219, 319, 293, 203 | 10.3(0.61-49.4) |
| 2-Methylerythritol | meso-erythritol | 219, 319, 293, 203 | 48.6(1.00-194) |
| C-5 alkene triols | meso-erythritol | 231, 147, 73 | 82.0(1.21-367) |
| *Monoterpene tracers* | | | |
| 3-Hydroxyglutaric acid | cis-ketopinic acid | 349, 275, 303, 185, 365 | 14.7(1.99-58.4) |
| *Biomass burning tracers* | | | |
| Levoglucosan | Levoglucosan | 333, 217, 204 | 18.7(3.62-101) |

Table S2. List of quantified Nitrogen aromatics compounds by LCMS with compound formula, surrogate compound, molecular weight (MW), quantitation ion and major product ions.

| Nitro-aromatics | Chemical Formula | Quantified as | MW | Quant $[M-H]^-$ ion (m/z) | Major $[M-H]^-$product ions(m/z) | Mean Concentration (Range) (ng/m$^3$) |
|---|---|---|---|---|---|---|
| Nitrophenol | $C_6H_5NO_3$ | 4-nitrophenol | 139.1 | 138.0196 | 108(-NO), 92(-$NO_2$) | 0.01(ND-0.06) |
| Nitrocatechol | $C_6H_5NO_4$ | 4-nitrocatechol | 155.1 | 154.0145 | 123,124(-NO), 95, 108(-$NO_2$) | 0.08(ND-1.26) |
| Methyl nitro catechol | $C_7H_7NO_4$ | 2-methyl-4-nitroresorcinol | 169.1 | 168.0301 | 138(-NO), 122(-$NO_2$) 109 | 0.02(ND-0.44) |
| Methyl nitro phenol | $C_7H_7NO_3$ | 2-methyl-4-nitrophenol | 153.1 | 152.0353 | 122(-NO), 106(-$NO_2$) | 0.00(ND-0.02) |
| Dimethyl nitro catechol | $C_8H_9NO_4$ | 2-methyl-4-nitroresorcinol | 183.2 | 182.0459 | 123, 152(-NO) | 0.01(ND-0.12) |

Table S3. List of quantified Organosulfates compounds by LCMS with compound formula, surrogate compound, molecular weight (MW), quantitation ion and major product ions.

| Organosulfates | Chemical Formula | Quantified as | MW | Quant $[M-H]^-$ ion (m/z) | Major $[M-H]^-$ product ions(m/z) | Mean Concentration (Range) ($ng/m^3$) |
|---|---|---|---|---|---|---|
| Isoprene SOA | $C_2H_4O_5S$ | Camphor sulfonic acid | 140.1 | 138.9707 | 97,80 | 0.51(0.06-1.41) |
| | $C_3H_6O_5S$ | Camphor sulfonic acid | 154.1 | 152.9863 | 97,80 | 3.02(0.30-9.75) |
| | $C_2H_4O_6S$ | Camphor sulfonic acid | 156.1 | 154.9656 | 97,80 | 7.24(0.11-28.4) |
| | $C_5H_{10}O_6S$ | Camphor sulfonic acid | 198.2 | 197.0125 | 97,80 | 0.81(0.06-3.89) |
| | $C_3H_6O_6S$ | Camphor sulfonic acid | 170.1 | 168.9812 | 97,80 | 0.85(0.01-3.67) |
| | $C_4H_8O_7S$ | Camphor sulfonic acid | 200.2 | 198.9918 | 119,97,80 | 3.62(ND-18.6) |
| | $C_5H_{12}O_7S$ | Camphor sulfonic acid | 216.2 | 215.0231 | 97,80 | 50.7(1.84-167) |
| | $C_5H_8O_7S$ | Camphor sulfonic acid | 212.2 | 210.9918 | 97,80 | 6.17(0.19-20.2) |
| | $C_5H_{10}O_7S$ | Camphor sulfonic acid | 214.2 | 213.0074 | 97,80 | 5.50(0.19-22.0) |
| | $C_5H_{11}NO_9S$ | Camphor sulfonic acid | 261.0 | 260.0082 | 197(-$HNO_3$), 97,80 | 5.95(0.31-28.8) |
| Monoterpene SOA | $C_{10}H_{18}O_5S$ | Camphor sulfonic acid | 250.3 | 249.0802 | 97,80 | 0.59(0.03-1.86) |
| | $C_{10}H_{16}O_7S$ | Camphor sulfonic acid | 280.3 | 279.0543 | 235(-$CO_2$), 97,80 | 2.07(0.33-7.92) |
| | $C_7H_{12}O_7S$ | Camphor sulfonic acid | 240.0 | 239.0231 | 97,159 | 2.55(0.49-7.69) |
| | $C_{10}H_{18}O_7S$ | Camphor sulfonic acid | 282.1 | 281.0700 | 97,80 | 1.30(0.02-6.35) |
| | $C_{10}H_{17}NO_7S$ | Camphor sulfonic acid | 295.3 | 294.0653 | 96,80, 220, 231(-$HNO_3$), 247(-HONO) | 1.51(0.11-14.6) |
| | $C_{10}H_{17}NO_8S$ | Camphor sulfonic acid | 311.1 | 310.0602 | 247(-$HNO_3$), 263(-HONO), 97,80 | 0.74(0.04-5.41) |
| | $C_{10}H_{17}NO_9S$ | Camphor sulfonic acid | 327.1 | 326.0551 | 279(-HONO), 263(-$HNO_3$), 97,80 | 0.48(0.02-4.30) |
| | $C_{10}H_{15}NO_9S$ | Camphor sulfonic acid | 325.0 | 324.0395 | 97,80,277(-HONO),165,119 | 0.14(ND-0.72) |
| | $C_{10}H_{17}NO_{10}S$ | Camphor sulfonic acid | 343.1 | 342.0500 | 97,80,279(-$HNO_3$),261,201,199 | 1.86(0.13-10.4) |
| Other SOA | $C_5H_{10}O_8S$ | Camphor sulfonic acid | 230.2 | 229.0024 | 149,97,80 | 1.42(ND-5.40) |

Table S4. List of quantified organic acids compounds by LCMS with compound formula, surrogate compound, molecular weight (MW), quantitation ion and major product ions.

| Terpenoic acids | Chemical Formula | Quantified as | MW | Quant $[M-H]^-$ ion (m/z) | Major $[M-H]^-$ product ions(m/z) | Mean Concentration (Range) ($ng/m^3$) |
|---|---|---|---|---|---|---|
| | $C_8H_{12}O_5$ | Suberic acid | 188.1 | 187.0617 | 125(-[$H_2O$+$CO_2$])<br>143(-$CO_2$) | 54.4(5.54-154) |
| | $C_7H_{10}O_5$ | Suberic acid | 174.1 | 173.0455 | 129(-$CO_2$)<br>111(-[$H_2O$+$CO_2$]) | 26.7(4.03-65.7) |
| | $C_8H_{12}O_6$ | Suberic acid | 204.1 | 203.0561 | 141(-[$H_2O$+$CO_2$])<br>185(-$H_2O$) | 22.3(2.27-60.4) |
| | $C_9H_{12}O_6$ | Suberic acid | 216.1 | 215.0573 | 171(-$CO_2$)<br>153(-[$H_2O$+$CO_2$]) | 2.82(0.02-12.4) |
| | $C_8H_{14}O_5$ | Suberic acid | 190.1 | 189.0768 | 145(-$CO_2$)<br>127(-[$H_2O$+$CO_2$]) | 4.58(ND-15.1) |
| | $C_8H_{10}O_5$ | Suberic acid | 186.1 | 185.0465 | 141(-$CO_2$)<br>125(-$CH_3COOH$) | 6.59(1.52-18.2) |
| | $C_8H_{12}O_4$ | Suberic acid | 172.1 | 171.0663 | 127(-$CO_2$) | 7.03(1.04-19.4) |
| | $C_7H_{10}O_6$ | Suberic acid | 190.1 | 189.0405 | 145(-$CO_2$)<br>127(-[$H_2O$+$CO_2$]) | 8.78(0.98-22.9) |
| | $C_5H_8O_4$ | Suberic acid | 132 | 131.035 | 87(-$CO_2$) | 2.88(0.54-9.05) |
| | $C_7H_{12}O_5$ | Suberic acid | 176.1 | 175.0612 | 113(-[$H_2O$+$CO_2$]) | 2.04(ND-6.95) |
| | $C_9H_{14}O_4$ | Suberic acid | 186.1 | 185.0819 | 141(-$CO_2$)<br>123(-[$H_2O$+$CO_2$]) | 2.82(0.02-9.21) |
| | $C_7H_{10}O_4$ | Suberic acid | 158.1 | 157.0506 | 113(-$CO_2$) | 5.01(0.68-17.7) |
| | $C_6H_8O_4$ | Suberic acid | 144 | 143.035 | 99(-$CO_2$)<br>83(-$CH_3COOH$) | 1.67(ND-5.30) |
| | $C_{10}H_{16}O_5$ | cis-Pinonic acid | 216.1 | 215.0925 | 171(-$CO_2$)<br>153(-[$H_2O$+$CO_2$]) | 45.3(10.5-163.4) |
| | $C_{10}H_{16}O_6$ | cis-Pinonic acid | 232.1 | 231.0874 | 169(-[$H_2O$+$CO_2$]) | 13.5(3.32-35.8) |
| | $C_{12}H_{18}O_5$ | Dodecanedioic acid | 242.1 | 241.1084 | 197(-$CO_2$) | 1.88(ND-6.32) |
| | $C_{13}H_{20}O_5$ | Dodecanedioic acid | 256.1 | 255.1247 | 211(-$CO_2$)<br>193(-[$H_2O$+$CO_2$]) | 4.51(0.59-13.68) |
| | $C_{10}H_{14}O_5$ | cis-Pinonic acid | 214.1 | 213.0768 | 169(-$CO_2$) | 63.9(15.0-216.4) |

Table S5. Recoveries of standard/surrogate compounds used in HPLC-ESI(-)-QTOF-MS and GCMS analysis.

| Standard Compound | Recovery (%) |
|---|---|
| 4-Nitrophenol | 97.0±0.7 |
| 4-Nitrocatechol | 75.2±5.6 |
| 2-Methyl-4-nitroresorcinol | 102.0±3.3 |
| 2-Methyl-4-nitrophenol | 98.3±2.2 |
| Camphor sulfonic acid | 89.2±1.4 |
| Suberic acid | 117.2±5.9 |
| cis-Pinonic acid | 129.4±4.2 |
| Dodecanedioic acid | 117.0±4.9 |
| meso-Erythritol | 88.5±3.2 |
| cis-Ketopinic acid | 95.3±1.7 |
| Levoglucosan | 98.6±1.4 |

Table S6. Correlation coefficients among nitro-aromatics and levoglucosan.

| | $C_6H_5NO_3$ | $C_6H_5NO_4$ | $C_7H_7NO_4$ | $C_7H_7NO_3$ | $C_8H_9NO_4$ | Levoglucosan |
|---|---|---|---|---|---|---|
| $C_6H_5NO_3$ | | **0.860** | **0.429** | **0.778** | **0.359** | **0.538** |
| $C_6H_5NO_4$ | | | **0.388** | **0.690** | 0.289 | **0.608** |
| $C_7H_7NO_4$ | | | | **0.488** | **0.674** | **0.545** |
| $C_7H_7NO_3$ | | | | | **0.467** | **0.460** |
| $C_8H_9NO_4$ | | | | | | **0.546** |
| Levoglucosan | | | | | | |

Values in bold indicate $p<0.01$

Table S7. Correlation coefficients among organosulfates and SOA tracers.

| m/z | OS 215 | OS 199 | OS 197 | NOS 260 | OS 279 | NOS 294 | 2-MG | C5-alkene triols | 2-methyl-threitol | 2-methyl-erythritol | HGA |
|---|---|---|---|---|---|---|---|---|---|---|---|
| OS 215 | | **0.696** | **0.861** | **0.829** | **0.484** | -0.217 | **0.591** | **0.974** | **0.896** | **0.932** | **0.699** |
| OS 199 | | | **0.753** | **0.784** | **0.634** | -0.015 | **0.686** | **0.759** | **0.715** | **0.721** | **0.700** |
| OS 197 | | | | **0.961** | **0.748** | 0.009 | **0.690** | **0.881** | **0.848** | **0.871** | **0.769** |
| NOS 260 | | | | | **0.800** | -0.057 | **0.760** | **0.846** | **0.829** | **0.832** | **0.823** |
| OS 279 | | | | | | 0.320 | **0.745** | **0.512** | **0.515** | **0.506** | **0.819** |
| NOS 294 | | | | | | | -0.061 | -0.162 | -0.195 | -0.147 | 0.029 |
| 2-MG | | | | | | | | **0.609** | **0.644** | **0.588** | **0.832** |
| C5-alkene triols | | | | | | | | | **0.934** | **0.968** | **0.747** |
| 2-methyl-threitol | | | | | | | | | | **0.975** | **0.747** |
| 2-methyl-erythritol | | | | | | | | | | | **0.740** |
| HGA | | | | | | | | | | | |

Values in bold indicate $p<0.01$; OS-organosulfate, NOS-nitrooxy organosulfate, 2-MG-2-Methylglyceric acid

Table S8. Correlation coefficients among terpenoic acids.

| m/z | 187 C8 | 173 C7 | 203 C8 | 215 C9 | 189 C7 | 185 C8 | 171 C8 | 189 C7 | 131 C5 | 175 C7 | 185 C9 | 157 C7 | 143 C6 | 215 C10 | 231 C10 | 241 C12 | 255 C13 | 213 C10 | HGA |
|---|---|---|---|---|---|---|---|---|---|---|---|---|---|---|---|---|---|---|---|
| 187C8 | | **0.988** | **0.959** | **0.857** | **0.916** | **0.935** | **0.879** | **0.952** | **0.696** | **0.925** | **0.675** | 0.259 | **0.788** | **0.867** | **0.831** | **0.883** | **0.697** | **0.861** | **0.926** |
| 173C7 | | | **0.972** | **0.847** | **0.913** | **0.955** | **0.882** | **0.968** | **0.743** | **0.918** | **0.676** | 0.320 | **0.810** | **0.894** | **0.866** | **0.875** | **0.713** | **0.891** | **0.932** |
| 203C8 | | | | **0.846** | **0.876** | **0.951** | **0.858** | **0.963** | **0.821** | **0.866** | **0.627** | 0.302 | **0.755** | **0.899** | **0.886** | **0.884** | **0.804** | **0.900** | **0.944** |
| 215C9 | | | | | **0.760** | **0.790** | **0.745** | **0.839** | **0.621** | **0.774** | **0.578** | 0.151 | **0.677** | **0.687** | **0.653** | **0.820** | **0.635** | **0.688** | **0.820** |
| 189C8 | | | | | | **0.887** | **0.929** | **0.857** | **0.597** | **0.973** | **0.846** | **0.463** | **0.811** | **0.919** | **0.751** | **0.868** | **0.648** | **0.899** | **0.851** |
| 185C8 | | | | | | | **0.886** | **0.917** | **0.740** | **0.888** | **0.690** | **0.378** | **0.731** | **0.912** | **0.868** | **0.815** | **0.703** | **0.921** | **0.881** |
| 171C8 | | | | | | | | **0.824** | **0.565** | **0.903** | **0.847** | **0.528** | **0.728** | **0.880** | **0.720** | **0.789** | **0.582** | **0.873** | **0.799** |
| 189C7 | | | | | | | | | **0.796** | **0.876** | **0.619** | 0.248 | **0.839** | **0.846** | **0.825** | **0.872** | **0.736** | **0.827** | **0.957** |
| 131C5 | | | | | | | | | | **0.595** | 0.334 | 0.229 | **0.640** | **0.758** | **0.814** | **0.666** | **0.802** | **0.741** | **0.827** |
| 175C7 | | | | | | | | | | | **0.813** | **0.381** | **0.854** | **0.888** | **0.732** | **0.846** | **0.599** | **0.849** | **0.859** |
| 185C9 | | | | | | | | | | | | **0.565** | **0.700** | **0.743** | **0.450** | **0.729** | **0.435** | **0.705** | **0.592** |
| 157C7 | | | | | | | | | | | | | **0.390** | **0.527** | 0.266 | 0.276 | 0.213 | **0.537** | 0.233 |
| 143C6 | | | | | | | | | | | | | | **0.745** | **0.597** | **0.778** | **0.568** | **0.695** | **0.807** |
| 215C10 | | | | | | | | | | | | | | | **0.867** | **0.828** | **0.750** | **0.969** | **0.852** |
| 231C10 | | | | | | | | | | | | | | | | **0.696** | **0.740** | **0.889** | **0.835** |
| 241C12 | | | | | | | | | | | | | | | | | **0.843** | **0.806** | **0.860** |
| 255C13 | | | | | | | | | | | | | | | | | | **0.752** | **0.760** |
| 213C10 | | | | | | | | | | | | | | | | | | | **0.829** |
| HGA* | | | | | | | | | | | | | | | | | | | |

*HGA: 3-hydroxyglutaric acid
Values in bold indicate $p<0.01$
Only m/z and carbon number are shown

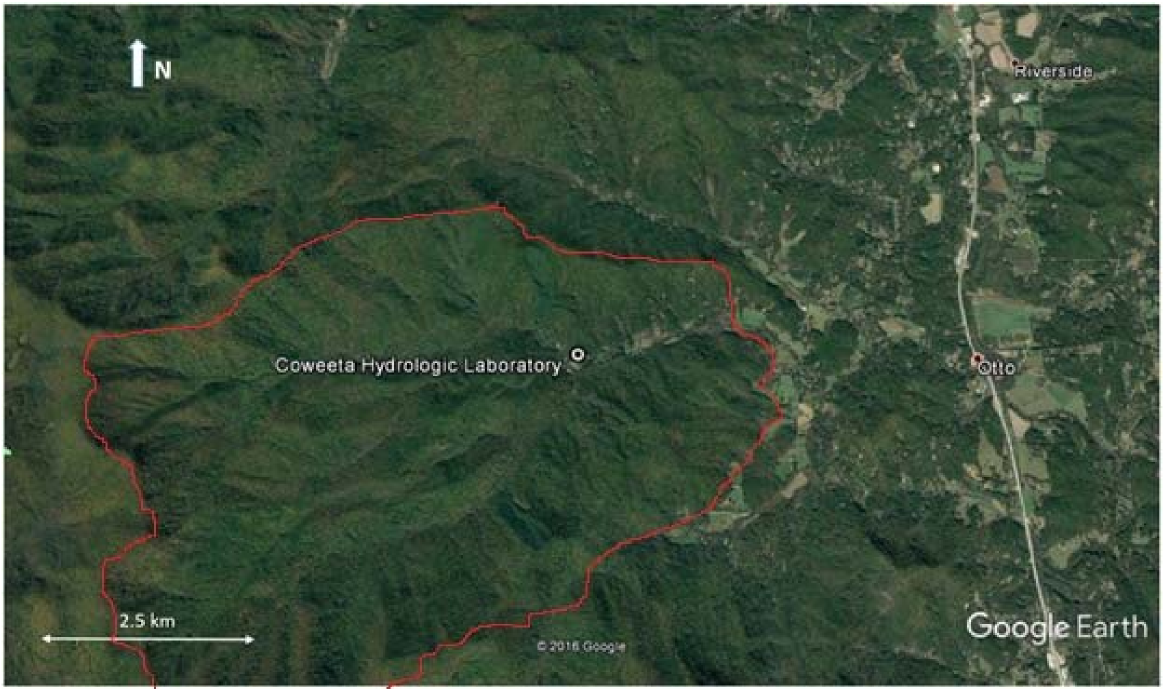


Figure S1. Satellite image of Coweeta sampling site (red outline represents the Coweeta basin).

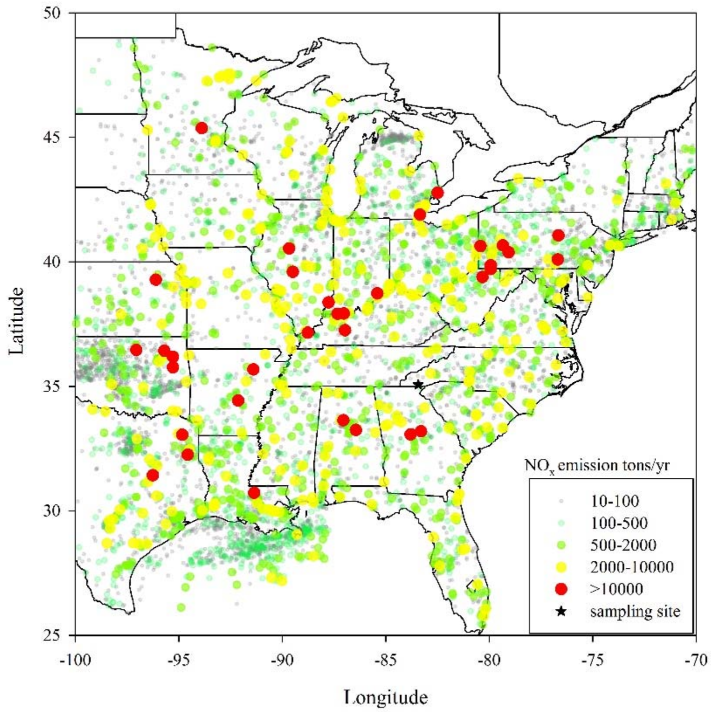


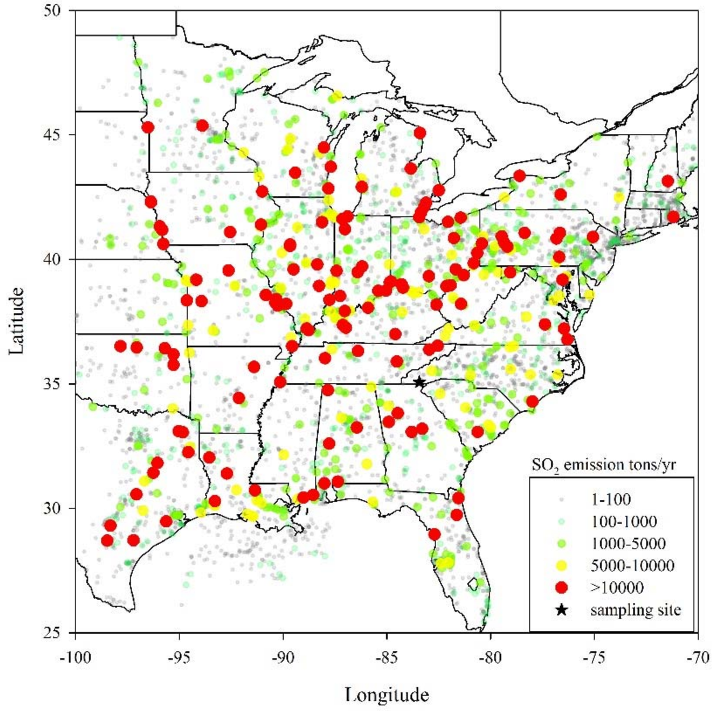


Figure S2. Emission inventories of NOx and $SO_2$ point sources in the eastern U.S. (2011 EPA National Emissions Inventory).

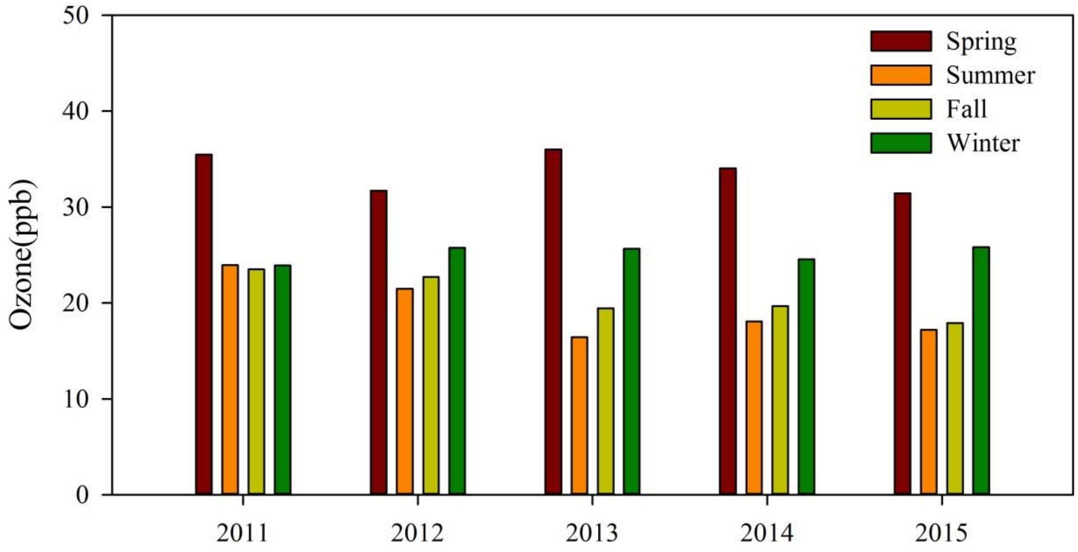


Figure S3. Seasonal ozone average concentrations from 2011 to 2015 at Coweeta sampling site (Spring: March, April and May; Summer: June, July and August; Fall: September, October and November; Winter: December, January and February).

## Back trajectory cluster analysis

Backward air mass trajectories were calculated for select periods using the Hybrid Single Particle Lagrangian Integrated Trajectory (HYSPLIT) model (Draxler and Rolph, 2003) with NOAA ARL EDAS 40 km meteorological data. Hourly trajectories for each sampling days were run for 72hr (120hr for regional biomass burning event towards end of October 2015) periods at an arriving height of 1000 m above the ground level. In order to better understand different synoptic circulation patterns, clusters of back trajectories were analyzed (Dorling et al., 1992; Dorling and Davies, 1995). The assignment of individual trajectories to a given cluster was carried out by minimizing the internal variability within the group of trajectories and maximizing the external variability between different groups based on the trajectory co-ordinates. Back trajectories and cluster analysis were computed by HYSPLIT 4. The number of clusters was determined by identifying a sudden change (criterion set at 30%) in the total spatial variance, which is the sum of all the cluster spatial variances.

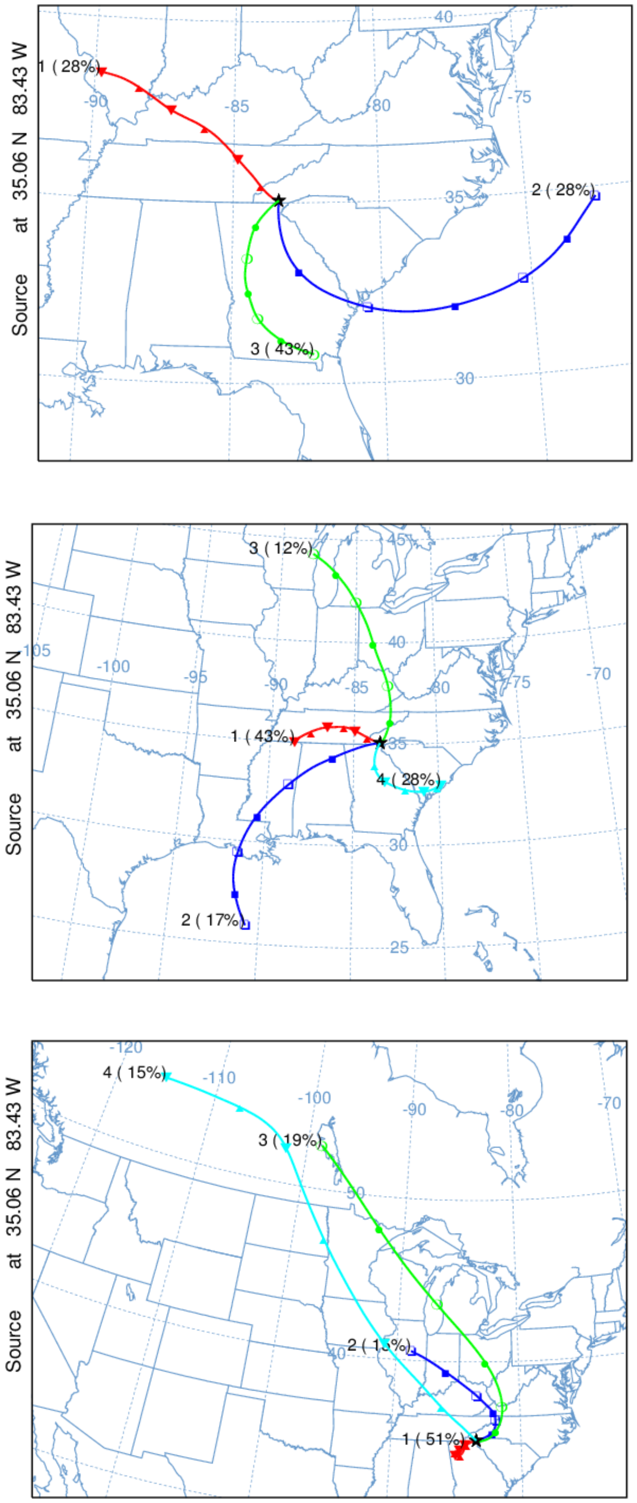


Figure S4. Back trajectory clusters for spring, summer and fall 2015(from top to bottom) at Coweeta for intensive sampling periods.

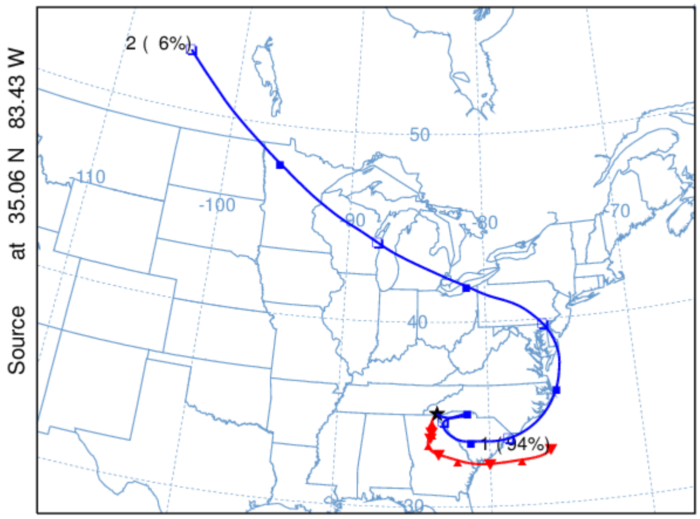


Figure S5. Back trajectory (120hr periods) clusters for regional biomass burning event on October 24th and 25th 2015 at Coweeta.

**PMF analysis and results**

To identify potential sources or processes related to particulate WSOC and WSON, bulk OC and EC, major inorganic ions ($NH_4^+$, $SO_4^{2-}$), organosulfates, nitro-aromatic compounds, terpenoic acids and levoglucosan were included for PMF analysis. Due to the small sample number, organosulfates, nitro-aromatic compounds and terpenoic acids were combined within each group based on the correlation of concentrations and potential sources. Among the 15 inputs species, two isoprene derived organosulfates and three monoterpene derived organosulfates are included. Iso_OS1 represents the combination of $C_5H_{10}O_6S$, $C_4H_8O_7S$ and $C_5H_{12}O_7S$; Iso_OS2 is $C_5H_{11}NO_9S$; Mono_OS1 is the sum of $C_{10}H_{18}O_5S$ and $C_{10}H_{16}O7S$; Mono_OS2 is composed of $C_{10}H_{17}NO_8S$ and $C_{10}H_{17}NO_9S$; Mono_OS3 is $C_{10}H_{17}NO_7S$; Iso_SOA includes methyltetrols, 2-methylglyceric acid and C-5 alkene triols; NitroArom and T_acids represent the sum of all nitro-aromatic compounds and terpenoic acids measured in this work, respectively. The uncertainties associated with each input species were estimated by:

$$unc = \sqrt{(20\% \times Conc.)^2 + (blk)^2}$$

where *Conc.* is the measured concentration of each species and *blk* is the average of the field blank measurements. Due to the low filed blank contamination, each input species has a signal to noise ratio higher than 3. The missing species observation was replaced by the geometric mean of the remaining observations, and the associated uncertainty was set as four times the geometric mean.

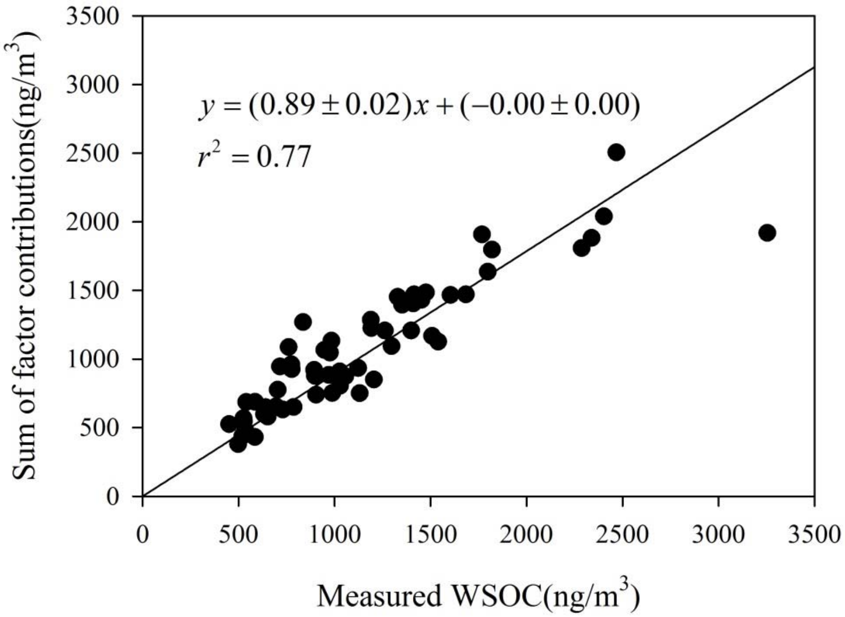


Figure S6. Linear regression between integrated PMF resolved factor contributions and measured WSOC.

**References**

Dorling, S.R., Davies, T.D., 1995. Extending cluster analysis–synoptic meteorology links to characterize chemical climates at six northwest European monitoring stations. Atmospheric Environment 29, 145–167.

Dorling, S.R., Davies, T.D., Pierce, C.E., 1992. Cluster analysis: a technique for estimating the synoptic meteorological controls on air and precipitation chemistry—method and applications. Atmospheric Environment 26, 2575–2581.

Draxler, R.R., Rolph, G.D., 2003. HYSPLIT (hybrid single particle Lagrangian integrated trajectory) model access via website (http://www.arl.noaa.gov/ready/hysplit4.html). NOAA Air Resources Laboratory, Silver Spring, MD.
